# Tetra­aqua­tetra­kis{μ_3_-3,3′-[(*E*,*E*)-ethane-1,2-diylbis(nitrilo­methyl­idyne)]benzene-1,2-diolato}octa­zinc(II) *N*,*N*-dimethyl­formamide hexa­solvate

**DOI:** 10.1107/S1600536809046923

**Published:** 2009-11-11

**Authors:** Qin-Juan Xu, Li-Rong Lin, Di Sun, Rong-Bin Huang, Lan-Sun Zheng

**Affiliations:** a Department of Chemistry, College of Chemistry and Chemical Engineering, Xiamen University, Xiamen 361005, People’s Republic of China; b State Key Laboratory for Physical Chemistry of Solid Surfaces, Xiamen University, Xiamen 361005, People’s Republic of China

## Abstract

The asymmetric unit of the title compound [Zn_8_(C_16_H_12_N_2_O_4_)_4_(H_2_O)_4_]·6C_3_H_7_NO, consists of eight Zn^II^ cations, four tetra­valent anionic ligands, *L* ^4−^ (*L* ^4−^ = 3,3′-(1*E*,1′*E*)-(ethane-1,2-diylbis(azan-1-yl-1-yl­idene))bis­(methan-1-yl-1-yl­idene)dibenzene-1,2-bis­(olate), four coordinated water mol­ecules and six *N*,*N*-dimethyl­formamide solvate mol­ecules. The coordination complex comprises an octa­nuclear Zn^II^ unit with its Zn^II^ centers coordinated in two discrete distorted square-pyramidal geometries. Four Zn^II^ atoms each coordinate to two nitro­gen atoms and two phenolate oxygen atoms from an individual *L* ^4−^ ligand and one coordinated water mol­ecule. The other four Zn^II^ atoms each bind to five phenolate oxygen atoms from three different *L* ^4−^ ligands. In the crystal structure, the Zn^II^ complex unit, coordinated water mol­ecules and dimethyl­formamide solvate mol­ecules are linked *via* O—H⋯O and C—H⋯O hydrogen bonds. Mol­ecules are connected by additional inter­molecular O—H⋯O and C—H⋯O hydrogen bonds, forming an extensive three dimensional framework.

## Related literature

For applications of Schiff base–metal compounds, see: Xu *et al.* (2007 ▶); Wu *et al.* (2006 ▶); Cametti *et al.* (2008 ▶); Shi *et al.* (2009 ▶); Dochnahl *et al.* (2006 ▶); Wu *et al.* (2007 ▶); Rai *et al.* (2009 ▶). For the luminescence properties of zinc complexes, see: Yu *et al.* (2007 ▶); Kaplunov *et al.* (2008 ▶). For the geometrical analysis of polyhedra with coordination number 5, see: Addison *et al.* (1984 ▶). For Zn and Cd complexes of similar flexible, multidentate Schiff base ligands, see: Sanmartín *et al.* (2000 ▶). For synthesis of the ligand, see: Casellato *et al.* (1991 ▶); Miyamoto *et al.* (2002 ▶). For other related structures, see: Eltayeb *et al.* (2008 ▶); Chen *et al.* (2009 ▶); Miyamoto *et al.* (2002 ▶).
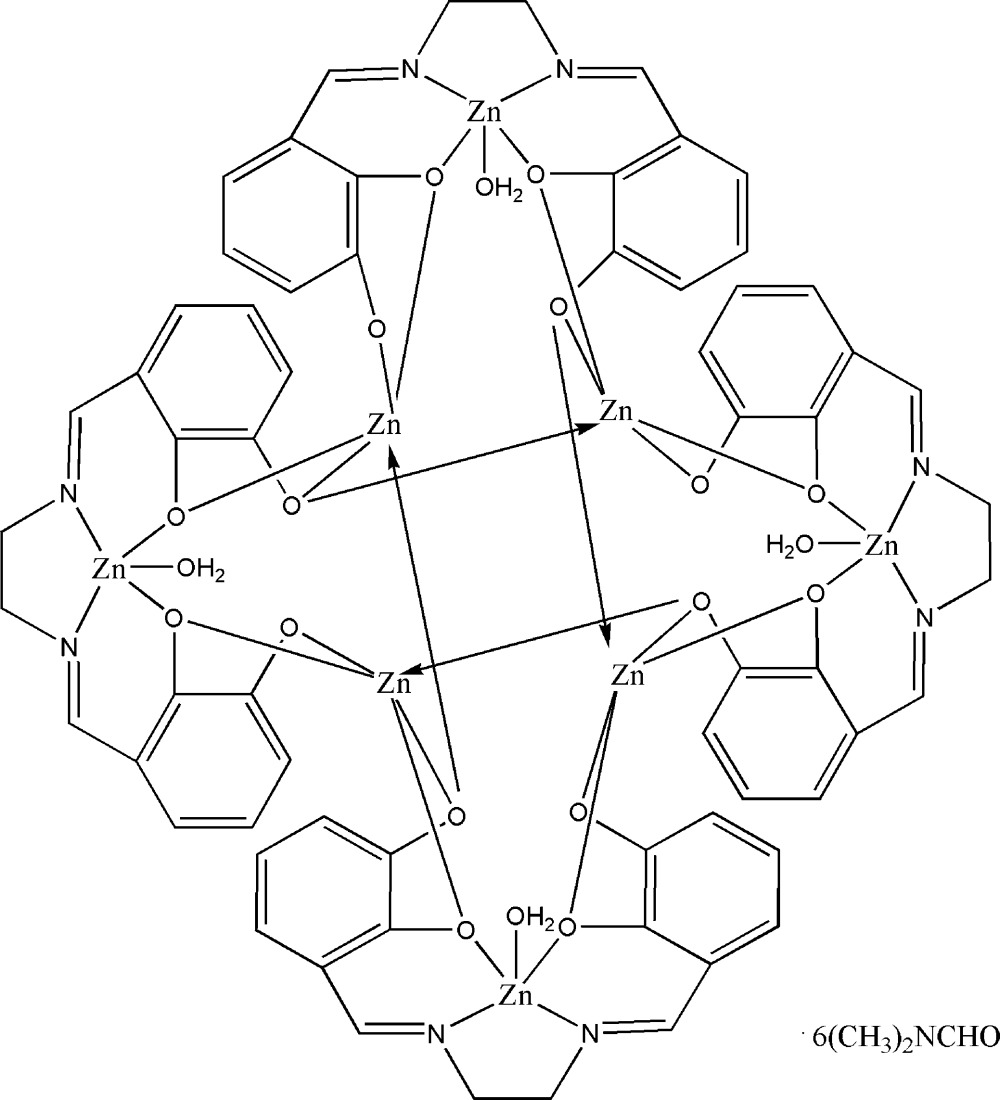


## Experimental

### 

#### Crystal data

[Zn_8_(C_16_H_12_N_2_O_4_)_4_(H_2_O)_4_]·6C_3_H_7_NO
*M* *_r_* = 2218.69
Triclinic, 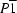

*a* = 13.5451 (4) Å
*b* = 14.2114 (4) Å
*c* = 25.0620 (6) Å
α = 80.599 (2)°
β = 76.519 (2)°
γ = 72.734 (2)°
*V* = 4456.9 (2) Å^3^
*Z* = 2
Mo *K*α radiation
μ = 2.20 mm^−1^
*T* = 298 K
0.31 × 0.20 × 0.15 mm

#### Data collection

Oxford Diffraction Gemini S Ultra diffractometer
Absorption correction: multi-scan (*CrysAlis RED*; Oxford Diffraction, 2008 ▶) *T*_min_ = 0.516, *T*_max_ = 0.719
51671 measured reflections
17430 independent reflections
13557 reflections with *I* > 2σ(*I*)
*R*_int_ = 0.027

#### Refinement

*R*[*F* ^2^ > 2σ(*F* ^2^)] = 0.030
*wR*(*F* ^2^) = 0.074
*S* = 1.04
17430 reflections
1184 parameters
H-atom parameters constrained
Δρ_max_ = 0.62 e Å^−3^
Δρ_min_ = −0.38 e Å^−3^

### 

Data collection: *CrysAlis CCD* (Oxford Diffraction, 2008 ▶); cell refinement: *CrysAlis RED* (Oxford Diffraction, 2008 ▶); data reduction: *CrysAlis RED*; program(s) used to solve structure: *SHELXS97* (Sheldrick, 2008 ▶); program(s) used to refine structure: *SHELXL97* (Sheldrick, 2008 ▶); molecular graphics: *DIAMOND* (Brandenburg 2008 ▶); software used to prepare material for publication: *SHELXL97* and *publCIF* (Westrip, 2009 ▶).

## Supplementary Material

Crystal structure: contains datablocks I, global. DOI: 10.1107/S1600536809046923/sj2663sup1.cif

Structure factors: contains datablocks I. DOI: 10.1107/S1600536809046923/sj2663Isup2.hkl

Additional supplementary materials:  crystallographic information; 3D view; checkCIF report

## Figures and Tables

**Table 1:** Hydrogen-bond geometry (Å, °)

*D*—H⋯*A*	*D*—H	H⋯*A*	*D*⋯*A*	*D*—H⋯*A*
O8*W*—H81*W*⋯O4	0.85	1.89	2.720 (3)	163
O8*W*—H82*W*⋯O2*A*	0.85	1.70	2.545 (3)	172
O7*W*—H21*W*⋯O5	0.85	1.84	2.677 (3)	167
O7*W*—H22*W*⋯O1*D*	0.85	1.76	2.604 (3)	173
O6*W*—H61*W*⋯O2	0.85	1.88	2.723 (3)	174
O6*W*—H62*W*⋯O2*C*	0.85	1.73	2.569 (3)	170
O5*W*—H51*W*⋯O6	0.85	1.89	2.731 (3)	171
O5*W*—H52*W*⋯O2*B*	0.85	1.77	2.612 (2)	171
C13*A*—H13*A*⋯O4^i^	0.93	2.52	3.339 (4)	148
C10*D*—H10*D*⋯O1	0.93	2.47	3.294 (4)	147
C13—H13⋯O4*B*	0.93	2.59	3.514 (4)	175

Symmetry code: (i) 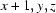.

**Fig. 1.**
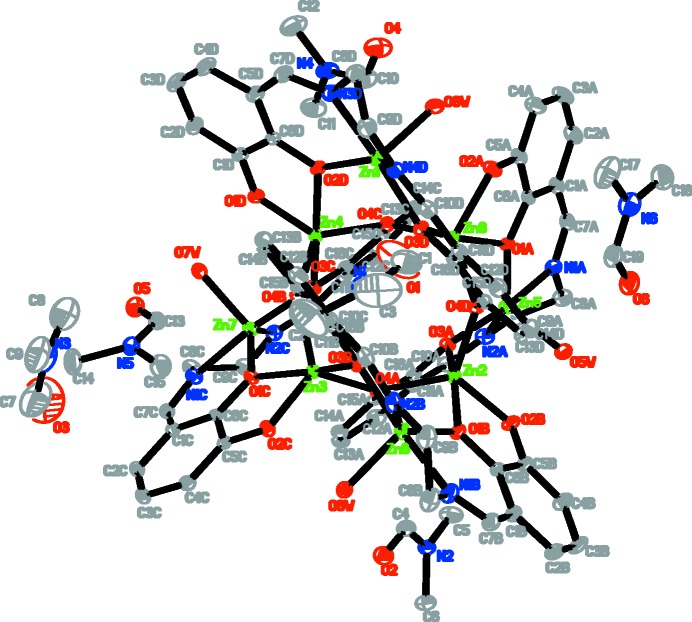
Structure of the title compound. with thermal ellipsoids drawn at 30% probability level. Hydrogen atoms are omitted for clarity.

**Fig. 2.**
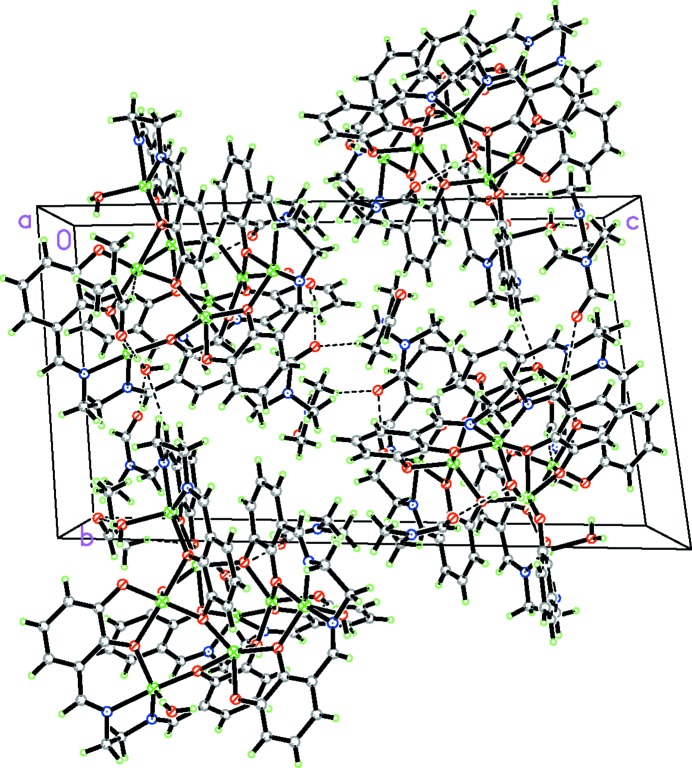
A perspective view of the three-dimensional supramolecular framework incorporating C–H···O, O–H···O hydrogen bonds viewed along b axis.

## supplementary crystallographic information

### Comment

Schiff base metal compounds have attracted great interest due to their significant potential applications in luminescent materials, ion recognition, biological processes and catalysis (Xu *et al.*, 2007; Wu *et al.*, 2006; Cametti *et al.*, 2008; Shi *et al.*, 2009; Dochnahl *et al.*, 2006; Wu *et al.*, 2007; Rai *et al.*, 2009). In particular, the luminescence properties of zinc compounds make them potentially useful as LED materials (Yu *et al.*, 2007; Kaplunov *et al.*, 2008). Herein we report the structure of an octanuclear zinc^II^ complex which exhibits strong fluorescence in the solid state.

The X-ray diffraction study shows that Zn^II^ cations are coordinated in two different distorted square-pyramidal geometries *viz*. ZnN_2_O_3_ and ZnO_5_. Four Zn^II^ cations (Zn5, Zn6, Zn7, Zn8) each coordinate to two N atoms and two O atoms from an *L*^4-^ which lie in the square plane; the O atom from a coordinated water molecule occupies the vertex position. These Zn—O and Zn—N bond lengths fall within the ranges 1.9821 (17)–2.0468 (18) Å and 2.055 (2)–2.102 (2) Å respectively. The other four inner Zn^II^ cations (Zn1, Zn2, Zn3, Zn4) are coordinated by five O atoms from three different *L*^4-^ ligands. For these the Zn—O bond lengths range from 1.9556 (17) to 2.1604 (18) Å. Distortion of the square pyramids is indicated by the calculated τ values (Addison *et al.*, 1984), the average of which are 0.4446 and 0.7089 for the ZnN_2_O_3_ and ZnO_5_ square-pyramids respectively (for an ideal square-pyramidal geometry, τ = 0). Flexibility, versatility and high *µ*-O bridging ability of the ligands are the crucial factors in forming the octanuclear unit (Sanmartín *et al.*, 2000). Bond lengths and angles in I fall in the normal ranges and are comparable with those for related structures (Eltayeb *et al.*, 2008; Chen *et al.*, 2009; Miyamoto *et al.*, 2002).

In the crystal structure intermolecular O—H···O, C—H···O hydrogen bonds among the water molecules, solvent molecules and the Zn(II) complex unit form an extensive three dimensional supramolecular structure.

### Experimental

The ligand 3,3'-(1E,1'E)-(ethane-1,2-diylbis(azan-1-yl-1-ylidene)) bis(methan-1-yl-1-ylidene)dibenzene-1,2-diol (*L)* was prepared according to the literature (Casellato *et al.*, 1991; Miyamoto *et al.*, 2002). The title compound (I) was synthesized by mixing *L* (0.030 g, 0.1 mmol) with zinc acetate dihydrate(0.047 g, 0.2 mmol) in DMF (15 mL). The solution was refluxed with stirring for 5 h, to yield a clear brown solution which was filtered and allowed to evaporate slowly at room temperature for two weeks to give brown block-shaped crystals of I.

### Refinement

All H atoms were placed in calculated positions and refined using a riding model [*C*—*H*(aryl) = 0.93 Å and *U*_iso_(H) = 1.2*U*_eq_(C); C—H(CH_3_) = 0.96 Å and *U*_iso_(H) = 1.5*U*_eq_(C); and O—H = 0.85 Å and *U*_iso_(H) = 1.2*U*_eq_(O)].

### Crystal data

**Table:**

[Zn_8_(C_16_H_12_N_2_O_4_)_4_(H_2_O)_4_]·6C_3_H_7_NO	*Z* = 2
*M**_r_* = 2218.69	*F*(000) = 2272
Triclinic, *P*1	*D*_x_ = 1.653 Mg m^−^^3^
Hall symbol: -P 1	Mo *K*α radiation, λ = 0.71073 Å
*a* = 13.5451 (4) Å	Cell parameters from 28634 reflections
*b* = 14.2114 (4) Å	θ = 2.2–29.6°
*c* = 25.0620 (6) Å	µ = 2.20 mm^−^^1^
α = 80.599 (2)°	*T* = 298 K
β = 76.519 (2)°	Block, brown
γ = 72.734 (2)°	0.31 × 0.20 × 0.15 mm
*V* = 4456.9 (2) Å^3^	

### Data collection

**Table:**

Oxford Diffraction Gemini S Ultra diffractometer	17430 independent reflections
Radiation source: sealed tube	13557 reflections with *I* > 2σ(*I*)
graphite	*R*_int_ = 0.027
Detector resolution: 16.1903 pixels mm^-1^	θ_max_ = 26.0°, θ_min_ = 2.2°
ω scans	*h* = −16→16
Absorption correction: multi-scan (*CrysAlis RED*; Oxford Diffraction, 2008)	*k* = −17→16
*T*_min_ = 0.516, *T*_max_ = 0.719	*l* = −29→30
51671 measured reflections	

### Refinement

**Table:**

Refinement on *F*^2^	Secondary atom site location: difference Fourier map
Least-squares matrix: full	Hydrogen site location: inferred from neighbouring sites
*R*[*F*^2^ > 2σ(*F*^2^)] = 0.030	H-atom parameters constrained
*wR*(*F*^2^) = 0.074	*w* = 1/[σ^2^(*F*_o_^2^) + (0.0409*P*)^2^] where *P* = (*F*_o_^2^ + 2*F*_c_^2^)/3
*S* = 1.03	(Δ/σ)_max_ = 0.003
17430 reflections	Δρ_max_ = 0.62 e Å^−^^3^
1184 parameters	Δρ_min_ = −0.38 e Å^−^^3^
0 restraints	Extinction correction: *SHELXL97* (Sheldrick, 2008), Fc^*^=kFc[1+0.001xFc^2^λ^3^/sin(2θ)]^-1/4^
Primary atom site location: structure-invariant direct methods	Extinction coefficient: 0.00041 (9)

### Special details

**Table:**

Geometry. All e.s.d.'s (except the e.s.d. in the dihedral angle between two l.s. planes) are estimated using the full covariance matrix. The cell e.s.d.'s are taken into account individually in the estimation of e.s.d.'s in distances, angles and torsion angles; correlations between e.s.d.'s in cell parameters are only used when they are defined by crystal symmetry. An approximate (isotropic) treatment of cell e.s.d.'s is used for estimating e.s.d.'s involving l.s. planes.
Refinement. Refinement of *F*^2^ against ALL reflections. The weighted *R*-factor *wR* and goodness of fit *S* are based on *F*^2^, conventional *R*-factors *R* are based on *F*, with *F* set to zero for negative *F*^2^. The threshold expression of *F*^2^ > σ(*F*^2^) is used only for calculating *R*-factors(gt) *etc*. and is not relevant to the choice of reflections for refinement. *R*-factors based on *F*^2^ are statistically about twice as large as those based on *F*, and *R*- factors based on ALL data will be even larger.

### Fractional atomic coordinates and isotropic or equivalent isotropic displacement parameters (Å^2^)

**Table:**

	*x*	*y*	*z*	*U*_iso_*/*U*_eq_	
Zn1	0.58891 (2)	0.10447 (2)	0.213483 (13)	0.01687 (8)	
Zn2	0.80680 (2)	0.19208 (2)	0.156595 (13)	0.01673 (8)	
Zn3	0.73308 (2)	0.34202 (2)	0.266806 (13)	0.01630 (8)	
Zn4	0.55372 (2)	0.20850 (2)	0.333987 (13)	0.01730 (8)	
Zn5	0.83945 (3)	−0.07182 (2)	0.184240 (14)	0.02050 (8)	
Zn6	0.73152 (3)	0.45407 (2)	0.127857 (13)	0.01912 (8)	
Zn7	0.77824 (3)	0.19745 (2)	0.392176 (13)	0.01933 (8)	
Zn8	0.33798 (2)	0.27824 (2)	0.262661 (13)	0.01999 (8)	
C1	0.2141 (3)	0.7241 (3)	0.08373 (17)	0.0499 (10)	
H1	0.1832	0.7099	0.0576	0.060*	
C1C	0.8622 (2)	0.40167 (19)	0.39310 (11)	0.0182 (6)	
C1B	0.9186 (2)	0.3732 (2)	0.01636 (12)	0.0241 (7)	
C1D	0.3966 (2)	0.2325 (2)	0.42770 (12)	0.0225 (6)	
C1A	0.6574 (2)	−0.1997 (2)	0.19311 (12)	0.0254 (7)	
C2	0.2501 (5)	0.8453 (4)	0.1272 (2)	0.0987 (19)	
H2E	0.2591	0.7931	0.1568	0.148*	
H2F	0.2049	0.9052	0.1418	0.148*	
H2G	0.3175	0.8555	0.1093	0.148*	
C2C	0.8892 (2)	0.48897 (19)	0.39659 (12)	0.0218 (6)	
H2C	0.9173	0.4929	0.4264	0.026*	
C2D	0.3449 (2)	0.2422 (2)	0.48217 (12)	0.0300 (7)	
H2D	0.3834	0.2203	0.5102	0.036*	
C2B	1.0036 (3)	0.3434 (2)	−0.02721 (13)	0.0316 (8)	
H2B	1.0202	0.3900	−0.0560	0.038*	
C2A	0.5831 (3)	−0.2559 (2)	0.20237 (14)	0.0350 (8)	
H2A	0.6069	−0.3241	0.2011	0.042*	
C3	0.1452 (4)	0.8978 (3)	0.0545 (2)	0.0874 (17)	
H3E	0.1126	0.8716	0.0324	0.131*	
H3F	0.1923	0.9329	0.0310	0.131*	
H3G	0.0918	0.9423	0.0780	0.131*	
C3C	0.8748 (2)	0.56829 (19)	0.35684 (12)	0.0229 (6)	
H3C	0.8921	0.6255	0.3599	0.027*	
C3B	1.0624 (3)	0.2467 (2)	−0.02790 (13)	0.0353 (8)	
H3B	1.1193	0.2280	−0.0566	0.042*	
C3D	0.2367 (3)	0.2839 (3)	0.49561 (14)	0.0388 (8)	
H3D	0.2033	0.2906	0.5322	0.047*	
C3A	0.4780 (3)	−0.2119 (2)	0.21299 (15)	0.0409 (9)	
H3A	0.4303	−0.2500	0.2197	0.049*	
C4	1.0482 (2)	0.3104 (2)	0.13947 (13)	0.0301 (7)	
H4	0.9875	0.2889	0.1518	0.036*	
C4C	0.8339 (2)	0.56161 (19)	0.31181 (12)	0.0215 (6)	
H4C	0.8254	0.6146	0.2845	0.026*	
C4A	0.4412 (3)	−0.1086 (2)	0.21383 (14)	0.0359 (8)	
H4A	0.3691	−0.0786	0.2210	0.043*	
C4B	1.0368 (3)	0.1767 (2)	0.01448 (13)	0.0323 (8)	
H4B	1.0771	0.1112	0.0137	0.039*	
C4D	0.1802 (3)	0.3147 (3)	0.45482 (14)	0.0375 (8)	
H4D	0.1080	0.3437	0.4639	0.045*	
C5	1.1462 (3)	0.1367 (2)	0.13910 (15)	0.0404 (9)	
H5E	1.0767	0.1273	0.1498	0.061*	
H5F	1.1853	0.1060	0.1677	0.061*	
H5G	1.1815	0.1071	0.1055	0.061*	
C5B	0.9529 (2)	0.2019 (2)	0.05793 (12)	0.0232 (6)	
C5C	0.8055 (2)	0.47775 (18)	0.30671 (11)	0.0161 (6)	
C5A	0.5113 (2)	−0.0506 (2)	0.20403 (13)	0.0270 (7)	
C5D	0.2282 (2)	0.3038 (2)	0.39866 (13)	0.0277 (7)	
C6	1.2372 (2)	0.2673 (2)	0.11138 (14)	0.0342 (8)	
H6E	1.2238	0.3380	0.1046	0.051*	
H6F	1.2735	0.2380	0.0778	0.051*	
H6G	1.2801	0.2430	0.1389	0.051*	
C6C	0.8181 (2)	0.39579 (18)	0.34848 (11)	0.0161 (6)	
C6D	0.3372 (2)	0.2611 (2)	0.38467 (12)	0.0212 (6)	
C6B	0.8935 (2)	0.30250 (19)	0.06029 (11)	0.0196 (6)	
C6A	0.6209 (2)	−0.09625 (19)	0.19530 (11)	0.0203 (6)	
C7	0.8913 (5)	0.3594 (7)	0.5800 (3)	0.135 (3)	
H7	0.9131	0.4168	0.5682	0.162*	
C7A	0.7665 (2)	−0.25372 (19)	0.18511 (12)	0.0252 (7)	
H7A	0.7820	−0.3218	0.1842	0.030*	
C7C	0.8937 (2)	0.3183 (2)	0.43252 (12)	0.0216 (6)	
H7C	0.9319	0.3256	0.4573	0.026*	
C7B	0.8577 (3)	0.4759 (2)	0.01193 (13)	0.0289 (7)	
H7B	0.8735	0.5148	−0.0208	0.035*	
C7D	0.1602 (2)	0.3422 (2)	0.35913 (13)	0.0309 (7)	
H7D	0.0900	0.3739	0.3725	0.037*	
C8	0.7623 (6)	0.2688 (6)	0.5940 (3)	0.158 (3)	
H8E	0.8204	0.2140	0.6018	0.237*	
H8F	0.7337	0.2544	0.5656	0.237*	
H8G	0.7088	0.2794	0.6268	0.237*	
C8A	0.9512 (2)	−0.2810 (2)	0.17584 (14)	0.0324 (8)	
H81A	0.9938	−0.2681	0.1400	0.039*	
H82A	0.9514	−0.3502	0.1811	0.039*	
C8D	0.1165 (2)	0.3862 (2)	0.27010 (13)	0.0282 (7)	
H81D	0.0510	0.4252	0.2904	0.034*	
H82D	0.1007	0.3381	0.2524	0.034*	
C8C	0.9247 (3)	0.1496 (2)	0.46949 (13)	0.0304 (7)	
H81C	0.8749	0.1336	0.5019	0.036*	
H82C	0.9822	0.1626	0.4814	0.036*	
C8B	0.7198 (3)	0.6175 (2)	0.04069 (13)	0.0345 (8)	
H81B	0.7336	0.6424	0.0021	0.041*	
H82B	0.7350	0.6605	0.0622	0.041*	
C9	0.7260 (4)	0.4416 (4)	0.5526 (2)	0.102 (2)	
H9E	0.7608	0.4926	0.5373	0.152*	
H9F	0.6669	0.4663	0.5811	0.152*	
H9G	0.7018	0.4221	0.5241	0.152*	
C9B	0.6063 (3)	0.6139 (2)	0.05900 (13)	0.0317 (7)	
H91B	0.5597	0.6805	0.0602	0.038*	
H92B	0.5878	0.5809	0.0333	0.038*	
C9A	0.9950 (3)	−0.2573 (2)	0.22101 (14)	0.0350 (8)	
H91A	0.9651	−0.2866	0.2565	0.042*	
H92A	1.0709	−0.2846	0.2146	0.042*	
C9C	0.9665 (2)	0.0644 (2)	0.43302 (13)	0.0290 (7)	
H91C	1.0264	0.0746	0.4051	0.035*	
H92C	0.9892	0.0022	0.4551	0.035*	
C9D	0.1704 (2)	0.4525 (2)	0.22754 (13)	0.0281 (7)	
H91D	0.1328	0.4759	0.1973	0.034*	
H92D	0.1713	0.5095	0.2439	0.034*	
C10	0.3447 (3)	0.0277 (3)	0.36853 (16)	0.0445 (9)	
H10	0.4072	0.0206	0.3426	0.053*	
C10D	0.3253 (2)	0.42027 (19)	0.15933 (12)	0.0224 (6)	
H10D	0.2889	0.4760	0.1397	0.027*	
C10C	0.8789 (2)	−0.0211 (2)	0.39456 (12)	0.0242 (6)	
H10C	0.9305	−0.0770	0.4042	0.029*	
C10A	1.0164 (2)	−0.1142 (2)	0.24711 (13)	0.0294 (7)	
H10A	1.0687	−0.1591	0.2637	0.035*	
C10B	0.5053 (2)	0.57722 (19)	0.14589 (12)	0.0237 (7)	
H10B	0.4503	0.6244	0.1324	0.028*	
C11	0.4513 (3)	−0.0297 (3)	0.43927 (16)	0.0503 (10)	
H11E	0.5082	−0.0379	0.4078	0.075*	
H11F	0.4554	−0.0918	0.4617	0.075*	
H11G	0.4563	0.0186	0.4605	0.075*	
C11A	0.9974 (2)	−0.0103 (2)	0.25361 (12)	0.0236 (6)	
C11B	0.4809 (2)	0.53101 (19)	0.20115 (12)	0.0203 (6)	
C11D	0.4305 (2)	0.37002 (19)	0.13263 (12)	0.0202 (6)	
C11C	0.8038 (2)	−0.03610 (19)	0.36708 (12)	0.0213 (6)	
C12	0.2590 (3)	0.0200 (3)	0.46356 (16)	0.0598 (11)	
H12E	0.1973	0.0434	0.4474	0.090*	
H12F	0.2605	0.0686	0.4856	0.090*	
H12G	0.2572	−0.0409	0.4863	0.090*	
C12B	0.3796 (2)	0.56990 (19)	0.23208 (13)	0.0255 (7)	
H12B	0.3321	0.6217	0.2158	0.031*	
C12D	0.4620 (2)	0.4048 (2)	0.07742 (12)	0.0245 (7)	
H12D	0.4159	0.4575	0.0609	0.029*	
C12A	1.0654 (2)	0.0127 (2)	0.28119 (13)	0.0284 (7)	
H12A	1.1183	−0.0381	0.2941	0.034*	
C12C	0.8095 (2)	−0.1356 (2)	0.36281 (12)	0.0257 (7)	
H12C	0.8605	−0.1863	0.3773	0.031*	
C13	0.5808 (2)	0.4794 (2)	0.40493 (15)	0.0328 (8)	
H13	0.5792	0.4447	0.3769	0.039*	
C13A	1.0554 (2)	0.1087 (2)	0.28957 (13)	0.0277 (7)	
H13A	1.1015	0.1229	0.3074	0.033*	
C13C	0.7414 (2)	−0.1588 (2)	0.33772 (12)	0.0274 (7)	
H13C	0.7449	−0.2246	0.3360	0.033*	
C13B	0.3494 (2)	0.5334 (2)	0.28531 (13)	0.0263 (7)	
H13B	0.2829	0.5613	0.3055	0.032*	
C13D	0.5593 (2)	0.3626 (2)	0.04734 (13)	0.0272 (7)	
H13D	0.5782	0.3849	0.0104	0.033*	
C14	0.5731 (3)	0.6339 (3)	0.43535 (15)	0.0557 (11)	
H14E	0.5744	0.5939	0.4701	0.084*	
H14F	0.5132	0.6909	0.4392	0.084*	
H14G	0.6366	0.6551	0.4239	0.084*	
C14D	0.6290 (2)	0.2861 (2)	0.07284 (12)	0.0225 (6)	
H14D	0.6953	0.2576	0.0527	0.027*	
C14B	0.4192 (2)	0.45412 (19)	0.30894 (12)	0.0234 (6)	
H14B	0.3987	0.4293	0.3453	0.028*	
C14A	0.9749 (2)	0.1844 (2)	0.27075 (12)	0.0233 (6)	
H14A	0.9677	0.2494	0.2762	0.028*	
C14C	0.6665 (2)	−0.0826 (2)	0.31476 (11)	0.0227 (6)	
H14C	0.6191	−0.0981	0.2984	0.027*	
C15	0.5546 (3)	0.6268 (2)	0.34034 (14)	0.0404 (9)	
H15E	0.5554	0.5802	0.3163	0.061*	
H15F	0.6119	0.6561	0.3256	0.061*	
H15G	0.4891	0.6776	0.3432	0.061*	
C15D	0.6021 (2)	0.25160 (18)	0.12719 (11)	0.0176 (6)	
C15A	0.9058 (2)	0.16483 (19)	0.24431 (12)	0.0191 (6)	
C15C	0.6618 (2)	0.01553 (19)	0.31594 (11)	0.0188 (6)	
C15B	0.5185 (2)	0.41096 (19)	0.27982 (11)	0.0180 (6)	
C16D	0.5004 (2)	0.29110 (18)	0.15854 (11)	0.0169 (6)	
C16A	0.9156 (2)	0.06531 (19)	0.23497 (11)	0.0182 (6)	
C16B	0.5528 (2)	0.45070 (18)	0.22506 (11)	0.0169 (6)	
C16C	0.7277 (2)	0.04129 (18)	0.34439 (11)	0.0185 (6)	
C17	0.4503 (3)	0.0572 (3)	0.08184 (18)	0.0706 (13)	
H17E	0.4588	0.1228	0.0777	0.106*	
H17F	0.4190	0.0407	0.1194	0.106*	
H17G	0.4055	0.0543	0.0579	0.106*	
C18	0.5566 (3)	−0.1142 (3)	0.06355 (16)	0.0559 (11)	
H18E	0.6274	−0.1497	0.0486	0.084*	
H18F	0.5105	−0.1153	0.0399	0.084*	
H18G	0.5342	−0.1450	0.0996	0.084*	
C19	0.6415 (3)	0.0134 (3)	0.06428 (14)	0.0427 (9)	
H19	0.6352	0.0777	0.0708	0.051*	
O1	0.2605 (3)	0.6555 (2)	0.11090 (17)	0.0970 (13)	
O8W	0.30266 (15)	0.15555 (14)	0.24940 (8)	0.0256 (5)	
H81W	0.2773	0.1277	0.2800	0.031*	
H82W	0.3584	0.1157	0.2339	0.031*	
O1D	0.50073 (15)	0.19540 (14)	0.41391 (8)	0.0220 (4)	
O1B	0.81689 (15)	0.32218 (12)	0.10517 (7)	0.0194 (4)	
O1A	0.68279 (14)	−0.03548 (12)	0.19083 (7)	0.0186 (4)	
O1C	0.78585 (14)	0.31941 (12)	0.34143 (7)	0.0180 (4)	
O2	1.03713 (16)	0.40089 (15)	0.13284 (9)	0.0334 (5)	
O7W	0.64676 (15)	0.23407 (14)	0.45143 (8)	0.0263 (5)	
H21W	0.6325	0.2950	0.4559	0.032*	
H22W	0.5955	0.2229	0.4417	0.032*	
O2A	0.47880 (15)	0.04808 (14)	0.20358 (9)	0.0282 (5)	
O2D	0.39208 (14)	0.24724 (13)	0.33367 (8)	0.0201 (4)	
O2B	0.92675 (15)	0.13516 (13)	0.09903 (8)	0.0246 (5)	
O2C	0.76505 (15)	0.47051 (12)	0.26465 (8)	0.0204 (4)	
O3D	0.47934 (14)	0.25003 (12)	0.21030 (7)	0.0178 (4)	
O6W	0.83069 (15)	0.50272 (13)	0.16033 (8)	0.0225 (4)	
H61W	0.8940	0.4698	0.1495	0.027*	
H62W	0.8146	0.4951	0.1953	0.027*	
O3B	0.65072 (14)	0.40850 (12)	0.20064 (7)	0.0181 (4)	
O3C	0.71186 (14)	0.13808 (12)	0.34707 (8)	0.0181 (4)	
O3A	0.84752 (14)	0.05388 (12)	0.20766 (8)	0.0184 (4)	
O3	0.9531 (4)	0.2817 (5)	0.6008 (3)	0.202 (3)	
O4	0.2608 (2)	0.0592 (2)	0.35162 (11)	0.0518 (7)	
O4D	0.66932 (14)	0.17732 (12)	0.15300 (7)	0.0185 (4)	
O5W	0.88746 (16)	−0.03509 (13)	0.10237 (8)	0.0276 (5)	
H51W	0.8385	−0.0308	0.0854	0.033*	
H52W	0.9022	0.0201	0.0978	0.033*	
O4B	0.58757 (14)	0.33431 (12)	0.30257 (7)	0.0178 (4)	
O4C	0.59075 (14)	0.09108 (13)	0.29267 (8)	0.0196 (4)	
O4A	0.83037 (14)	0.23753 (12)	0.22337 (8)	0.0185 (4)	
O5	0.59684 (18)	0.43137 (16)	0.44929 (10)	0.0377 (6)	
O6	0.7304 (2)	−0.04288 (19)	0.05325 (10)	0.0520 (7)	
N1	0.2031 (2)	0.8182 (2)	0.08764 (14)	0.0470 (8)	
N1B	0.7843 (2)	0.51637 (16)	0.04943 (10)	0.0261 (6)	
N1A	0.84363 (19)	−0.21777 (16)	0.17922 (10)	0.0242 (6)	
N1C	0.87322 (19)	0.23543 (17)	0.43600 (10)	0.0240 (5)	
N2B	0.5950 (2)	0.55929 (16)	0.11393 (10)	0.0229 (5)	
N2A	0.96830 (19)	−0.14941 (16)	0.22094 (11)	0.0275 (6)	
N2C	0.88149 (19)	0.06168 (16)	0.40698 (10)	0.0228 (5)	
N2	1.13880 (19)	0.24177 (17)	0.13052 (10)	0.0254 (6)	
N3D	0.18858 (18)	0.33626 (17)	0.30733 (10)	0.0250 (6)	
N3	0.7987 (3)	0.3572 (4)	0.57561 (17)	0.0816 (13)	
N4	0.3513 (2)	0.0037 (2)	0.42079 (12)	0.0363 (7)	
N4D	0.27811 (18)	0.39471 (16)	0.20743 (10)	0.0216 (5)	
N5	0.5657 (2)	0.57631 (19)	0.39416 (11)	0.0329 (6)	
N6	0.5530 (2)	−0.0133 (2)	0.06739 (12)	0.0448 (8)	

### Atomic displacement parameters (Å^2^)

**Table:**

	*U*^11^	*U*^22^	*U*^33^	*U*^12^	*U*^13^	*U*^23^
Zn1	0.01684 (17)	0.01506 (15)	0.01851 (19)	−0.00493 (12)	−0.00191 (13)	−0.00222 (12)
Zn2	0.01680 (17)	0.01434 (15)	0.01752 (19)	−0.00437 (13)	−0.00026 (13)	−0.00150 (12)
Zn3	0.01629 (17)	0.01574 (15)	0.01695 (19)	−0.00405 (13)	−0.00349 (13)	−0.00203 (12)
Zn4	0.01506 (17)	0.01794 (15)	0.01865 (19)	−0.00449 (13)	−0.00190 (13)	−0.00314 (12)
Zn5	0.01959 (18)	0.01326 (15)	0.0263 (2)	−0.00348 (13)	−0.00193 (14)	−0.00097 (13)
Zn6	0.02454 (19)	0.01410 (15)	0.01618 (19)	−0.00227 (13)	−0.00298 (14)	−0.00138 (12)
Zn7	0.02089 (18)	0.01696 (16)	0.0218 (2)	−0.00664 (13)	−0.00869 (14)	0.00268 (13)
Zn8	0.01472 (17)	0.02301 (17)	0.0203 (2)	−0.00227 (13)	−0.00264 (14)	−0.00327 (13)
C1	0.037 (2)	0.047 (2)	0.066 (3)	−0.0106 (18)	−0.016 (2)	0.0011 (19)
C1C	0.0133 (14)	0.0202 (13)	0.0208 (17)	−0.0032 (11)	−0.0024 (12)	−0.0049 (11)
C1B	0.0337 (18)	0.0196 (14)	0.0179 (17)	−0.0101 (13)	0.0001 (13)	−0.0008 (11)
C1D	0.0219 (16)	0.0244 (15)	0.0214 (18)	−0.0096 (12)	−0.0009 (13)	−0.0018 (12)
C1A	0.0319 (18)	0.0202 (14)	0.0269 (18)	−0.0092 (13)	−0.0063 (14)	−0.0058 (12)
C2	0.120 (5)	0.066 (3)	0.128 (5)	−0.033 (3)	−0.057 (4)	0.003 (3)
C2C	0.0179 (15)	0.0229 (14)	0.0262 (18)	−0.0038 (12)	−0.0054 (13)	−0.0085 (12)
C2D	0.0290 (18)	0.0436 (18)	0.0174 (18)	−0.0106 (15)	−0.0029 (14)	−0.0040 (13)
C2B	0.040 (2)	0.0266 (16)	0.0252 (19)	−0.0158 (15)	0.0074 (15)	−0.0006 (13)
C2A	0.041 (2)	0.0242 (16)	0.045 (2)	−0.0138 (15)	−0.0103 (17)	−0.0077 (14)
C3	0.067 (3)	0.060 (3)	0.116 (5)	−0.003 (2)	−0.035 (3)	0.041 (3)
C3C	0.0249 (16)	0.0162 (13)	0.0288 (18)	−0.0065 (12)	−0.0031 (13)	−0.0071 (12)
C3B	0.037 (2)	0.0340 (17)	0.026 (2)	−0.0114 (15)	0.0153 (15)	−0.0071 (14)
C3D	0.033 (2)	0.060 (2)	0.0200 (19)	−0.0145 (17)	0.0055 (15)	−0.0075 (16)
C3A	0.042 (2)	0.0313 (18)	0.059 (3)	−0.0214 (16)	−0.0106 (18)	−0.0107 (16)
C4	0.0274 (18)	0.0356 (18)	0.029 (2)	−0.0120 (14)	−0.0052 (14)	−0.0005 (14)
C4C	0.0220 (16)	0.0178 (13)	0.0226 (17)	−0.0049 (12)	−0.0021 (13)	−0.0004 (11)
C4A	0.0257 (18)	0.0320 (17)	0.053 (2)	−0.0092 (14)	−0.0079 (16)	−0.0084 (15)
C4B	0.0343 (19)	0.0211 (15)	0.032 (2)	−0.0050 (13)	0.0094 (15)	−0.0045 (13)
C4D	0.0198 (17)	0.055 (2)	0.030 (2)	−0.0030 (15)	0.0053 (15)	−0.0114 (16)
C5	0.032 (2)	0.0316 (18)	0.056 (3)	−0.0118 (15)	−0.0036 (17)	0.0002 (16)
C5B	0.0251 (17)	0.0206 (14)	0.0214 (17)	−0.0079 (12)	0.0044 (13)	−0.0050 (12)
C5C	0.0103 (14)	0.0185 (13)	0.0179 (16)	−0.0019 (11)	−0.0003 (11)	−0.0047 (11)
C5A	0.0292 (18)	0.0219 (15)	0.0337 (19)	−0.0092 (13)	−0.0048 (14)	−0.0114 (13)
C5D	0.0200 (16)	0.0348 (17)	0.0265 (19)	−0.0061 (13)	−0.0005 (13)	−0.0064 (13)
C6	0.0275 (18)	0.0346 (17)	0.038 (2)	−0.0122 (15)	−0.0004 (15)	0.0000 (14)
C6C	0.0097 (13)	0.0161 (13)	0.0216 (16)	−0.0033 (10)	0.0002 (11)	−0.0043 (11)
C6D	0.0175 (15)	0.0260 (15)	0.0208 (17)	−0.0098 (12)	0.0017 (12)	−0.0052 (12)
C6B	0.0225 (16)	0.0189 (13)	0.0176 (16)	−0.0092 (12)	0.0016 (12)	−0.0037 (11)
C6A	0.0252 (16)	0.0188 (13)	0.0195 (16)	−0.0097 (12)	−0.0032 (12)	−0.0043 (11)
C7	0.051 (4)	0.236 (9)	0.115 (6)	−0.004 (5)	−0.021 (4)	−0.066 (6)
C7A	0.0351 (18)	0.0125 (13)	0.0280 (18)	−0.0057 (13)	−0.0044 (14)	−0.0059 (12)
C7C	0.0189 (15)	0.0278 (15)	0.0198 (17)	−0.0059 (12)	−0.0069 (12)	−0.0035 (12)
C7B	0.042 (2)	0.0240 (15)	0.0207 (18)	−0.0146 (14)	−0.0006 (15)	0.0016 (12)
C7D	0.0165 (16)	0.0416 (18)	0.031 (2)	−0.0036 (14)	0.0010 (14)	−0.0103 (15)
C8	0.143 (7)	0.171 (8)	0.156 (8)	−0.056 (6)	0.042 (6)	−0.086 (6)
C8A	0.0309 (19)	0.0172 (14)	0.042 (2)	−0.0008 (13)	−0.0008 (15)	−0.0034 (13)
C8D	0.0162 (16)	0.0365 (17)	0.0294 (19)	−0.0021 (13)	−0.0042 (13)	−0.0059 (14)
C8C	0.041 (2)	0.0262 (16)	0.030 (2)	−0.0131 (14)	−0.0216 (16)	0.0090 (13)
C8B	0.054 (2)	0.0173 (15)	0.0232 (19)	−0.0037 (15)	−0.0033 (16)	0.0047 (12)
C9	0.071 (4)	0.145 (5)	0.079 (4)	0.014 (4)	−0.024 (3)	−0.050 (4)
C9B	0.042 (2)	0.0244 (16)	0.0246 (19)	−0.0006 (14)	−0.0136 (15)	0.0031 (13)
C9A	0.0322 (19)	0.0161 (14)	0.051 (2)	−0.0008 (13)	−0.0101 (16)	0.0045 (14)
C9C	0.0285 (18)	0.0253 (15)	0.034 (2)	−0.0038 (13)	−0.0197 (15)	0.0076 (13)
C9D	0.0206 (16)	0.0250 (15)	0.036 (2)	0.0030 (12)	−0.0080 (14)	−0.0093 (13)
C10	0.036 (2)	0.053 (2)	0.040 (2)	−0.0151 (18)	−0.0045 (18)	0.0066 (17)
C10D	0.0228 (16)	0.0161 (13)	0.0296 (19)	−0.0015 (12)	−0.0129 (14)	−0.0014 (12)
C10C	0.0249 (17)	0.0192 (14)	0.0242 (18)	−0.0020 (12)	−0.0054 (13)	0.0033 (12)
C10A	0.0224 (17)	0.0218 (15)	0.038 (2)	−0.0007 (13)	−0.0056 (14)	0.0039 (13)
C10B	0.0270 (17)	0.0161 (13)	0.0308 (19)	−0.0014 (12)	−0.0163 (14)	−0.0031 (12)
C11	0.041 (2)	0.060 (2)	0.052 (3)	−0.0193 (19)	−0.0200 (19)	0.0124 (19)
C11A	0.0196 (16)	0.0253 (15)	0.0220 (17)	−0.0044 (12)	−0.0015 (13)	0.0024 (12)
C11B	0.0209 (16)	0.0163 (13)	0.0272 (18)	−0.0047 (11)	−0.0082 (13)	−0.0076 (12)
C11D	0.0212 (16)	0.0179 (13)	0.0231 (17)	−0.0065 (12)	−0.0060 (12)	−0.0020 (11)
C11C	0.0227 (16)	0.0207 (14)	0.0174 (16)	−0.0053 (12)	−0.0009 (12)	0.0016 (11)
C12	0.041 (2)	0.086 (3)	0.045 (3)	−0.020 (2)	0.002 (2)	0.004 (2)
C12B	0.0185 (16)	0.0147 (13)	0.045 (2)	0.0004 (11)	−0.0129 (14)	−0.0066 (13)
C12D	0.0270 (17)	0.0241 (15)	0.0235 (18)	−0.0052 (13)	−0.0117 (14)	0.0010 (12)
C12A	0.0187 (16)	0.0304 (16)	0.033 (2)	−0.0030 (13)	−0.0091 (14)	0.0032 (13)
C12C	0.0325 (18)	0.0171 (14)	0.0233 (18)	−0.0014 (12)	−0.0064 (14)	0.0017 (12)
C13	0.0215 (17)	0.0371 (18)	0.042 (2)	−0.0062 (14)	−0.0009 (15)	−0.0205 (16)
C13A	0.0191 (16)	0.0348 (17)	0.0322 (19)	−0.0110 (13)	−0.0061 (14)	−0.0032 (14)
C13C	0.0398 (19)	0.0170 (14)	0.0246 (18)	−0.0087 (13)	−0.0038 (14)	−0.0019 (12)
C13B	0.0186 (16)	0.0210 (14)	0.038 (2)	−0.0027 (12)	−0.0006 (14)	−0.0116 (13)
C13D	0.0332 (19)	0.0317 (16)	0.0177 (17)	−0.0109 (14)	−0.0071 (14)	0.0017 (12)
C14	0.085 (3)	0.044 (2)	0.041 (2)	−0.025 (2)	0.002 (2)	−0.0200 (18)
C14D	0.0224 (16)	0.0260 (15)	0.0192 (17)	−0.0074 (12)	−0.0019 (13)	−0.0036 (12)
C14B	0.0237 (16)	0.0204 (14)	0.0259 (18)	−0.0067 (12)	0.0001 (13)	−0.0076 (12)
C14A	0.0194 (16)	0.0234 (14)	0.0274 (18)	−0.0080 (12)	−0.0017 (13)	−0.0031 (12)
C14C	0.0287 (17)	0.0230 (14)	0.0189 (17)	−0.0125 (13)	−0.0023 (13)	−0.0021 (12)
C15	0.034 (2)	0.0408 (19)	0.045 (2)	−0.0025 (16)	−0.0109 (17)	−0.0117 (16)
C15D	0.0199 (15)	0.0171 (13)	0.0169 (16)	−0.0061 (11)	−0.0044 (12)	−0.0014 (11)
C15A	0.0112 (14)	0.0207 (14)	0.0215 (17)	−0.0030 (11)	0.0014 (12)	−0.0008 (11)
C15C	0.0181 (15)	0.0197 (14)	0.0146 (16)	−0.0045 (11)	0.0025 (12)	0.0001 (11)
C15B	0.0173 (15)	0.0178 (13)	0.0203 (17)	−0.0050 (11)	−0.0035 (12)	−0.0054 (11)
C16D	0.0190 (15)	0.0178 (13)	0.0166 (16)	−0.0059 (11)	−0.0062 (12)	−0.0035 (11)
C16A	0.0141 (14)	0.0225 (14)	0.0164 (16)	−0.0069 (11)	0.0012 (11)	−0.0004 (11)
C16B	0.0176 (15)	0.0136 (12)	0.0226 (17)	−0.0051 (11)	−0.0056 (12)	−0.0069 (11)
C16C	0.0204 (15)	0.0162 (13)	0.0159 (16)	−0.0065 (11)	0.0014 (12)	0.0026 (11)
C17	0.053 (3)	0.084 (3)	0.049 (3)	0.022 (2)	−0.012 (2)	−0.009 (2)
C18	0.069 (3)	0.057 (2)	0.047 (3)	−0.021 (2)	−0.016 (2)	−0.0057 (19)
C19	0.048 (2)	0.040 (2)	0.037 (2)	−0.0070 (18)	−0.0011 (18)	−0.0144 (16)
O1	0.103 (3)	0.0353 (16)	0.165 (4)	−0.0102 (17)	−0.084 (3)	0.0227 (19)
O8W	0.0199 (11)	0.0258 (10)	0.0300 (13)	−0.0074 (9)	−0.0011 (9)	−0.0033 (9)
O1D	0.0172 (11)	0.0290 (10)	0.0200 (12)	−0.0073 (8)	−0.0032 (8)	−0.0018 (8)
O1B	0.0225 (11)	0.0149 (9)	0.0169 (11)	−0.0042 (8)	0.0033 (8)	−0.0019 (7)
O1A	0.0189 (10)	0.0165 (9)	0.0197 (11)	−0.0063 (8)	0.0003 (8)	−0.0033 (7)
O1C	0.0216 (11)	0.0155 (9)	0.0198 (11)	−0.0070 (8)	−0.0083 (8)	0.0000 (7)
O2	0.0306 (13)	0.0307 (12)	0.0384 (14)	−0.0079 (10)	−0.0061 (10)	−0.0040 (10)
O7W	0.0264 (12)	0.0328 (11)	0.0251 (12)	−0.0118 (9)	−0.0072 (9)	−0.0073 (9)
O2A	0.0198 (11)	0.0222 (10)	0.0452 (14)	−0.0046 (9)	−0.0061 (10)	−0.0130 (9)
O2D	0.0144 (10)	0.0270 (10)	0.0177 (11)	−0.0042 (8)	−0.0011 (8)	−0.0047 (8)
O2B	0.0249 (11)	0.0158 (9)	0.0261 (12)	−0.0048 (8)	0.0080 (9)	−0.0022 (8)
O2C	0.0282 (11)	0.0161 (9)	0.0189 (11)	−0.0072 (8)	−0.0082 (9)	0.0001 (8)
O3D	0.0179 (10)	0.0179 (9)	0.0155 (11)	−0.0039 (8)	−0.0016 (8)	−0.0002 (7)
O6W	0.0273 (11)	0.0196 (10)	0.0193 (12)	−0.0060 (8)	−0.0020 (9)	−0.0023 (8)
O3B	0.0168 (10)	0.0165 (9)	0.0183 (11)	−0.0004 (8)	−0.0033 (8)	−0.0021 (7)
O3C	0.0197 (10)	0.0138 (9)	0.0211 (11)	−0.0042 (8)	−0.0063 (8)	−0.0002 (7)
O3A	0.0165 (10)	0.0158 (9)	0.0229 (12)	−0.0045 (8)	−0.0047 (8)	−0.0002 (8)
O3	0.104 (4)	0.224 (7)	0.270 (8)	0.043 (4)	−0.106 (5)	−0.061 (6)
O4	0.0406 (16)	0.0706 (18)	0.0457 (17)	−0.0214 (14)	−0.0199 (13)	0.0188 (13)
O4D	0.0149 (10)	0.0179 (9)	0.0193 (11)	−0.0019 (8)	−0.0019 (8)	0.0010 (7)
O5W	0.0310 (12)	0.0202 (10)	0.0293 (13)	−0.0089 (9)	0.0031 (10)	−0.0051 (8)
O4B	0.0172 (10)	0.0161 (9)	0.0190 (11)	−0.0035 (8)	−0.0037 (8)	−0.0005 (7)
O4C	0.0223 (11)	0.0174 (9)	0.0187 (11)	−0.0046 (8)	−0.0040 (8)	−0.0021 (8)
O4A	0.0173 (10)	0.0148 (9)	0.0222 (11)	−0.0016 (8)	−0.0039 (8)	−0.0034 (8)
O5	0.0405 (15)	0.0359 (13)	0.0365 (16)	−0.0113 (11)	0.0006 (12)	−0.0125 (11)
O6	0.0431 (17)	0.0649 (17)	0.0472 (18)	−0.0088 (14)	−0.0011 (13)	−0.0249 (13)
N1	0.0401 (19)	0.0296 (16)	0.068 (2)	−0.0043 (13)	−0.0206 (16)	0.0076 (14)
N1B	0.0364 (16)	0.0187 (12)	0.0188 (15)	−0.0052 (11)	−0.0029 (12)	0.0030 (10)
N1A	0.0258 (14)	0.0132 (11)	0.0294 (15)	−0.0022 (10)	−0.0003 (11)	−0.0031 (10)
N1C	0.0273 (14)	0.0236 (13)	0.0240 (15)	−0.0087 (11)	−0.0124 (11)	0.0033 (10)
N2B	0.0314 (15)	0.0176 (11)	0.0189 (14)	−0.0017 (10)	−0.0106 (11)	−0.0006 (9)
N2A	0.0238 (14)	0.0176 (12)	0.0377 (17)	−0.0030 (10)	−0.0060 (12)	0.0017 (11)
N2C	0.0254 (14)	0.0216 (12)	0.0203 (14)	−0.0045 (10)	−0.0089 (11)	0.0044 (10)
N2	0.0214 (14)	0.0257 (13)	0.0288 (16)	−0.0089 (11)	−0.0018 (11)	−0.0022 (10)
N3D	0.0171 (13)	0.0308 (13)	0.0262 (16)	−0.0040 (11)	−0.0040 (11)	−0.0058 (11)
N3	0.047 (3)	0.127 (4)	0.063 (3)	0.000 (3)	−0.003 (2)	−0.043 (3)
N4	0.0301 (16)	0.0447 (16)	0.0319 (18)	−0.0144 (13)	−0.0062 (13)	0.0107 (13)
N4D	0.0153 (13)	0.0216 (12)	0.0271 (16)	−0.0025 (10)	−0.0035 (11)	−0.0055 (10)
N5	0.0306 (16)	0.0328 (15)	0.0346 (18)	−0.0089 (12)	0.0034 (13)	−0.0150 (12)
N6	0.0412 (19)	0.0437 (17)	0.042 (2)	0.0008 (14)	−0.0090 (15)	−0.0052 (14)

### Geometric parameters (Å, °)

**Table:**

Zn1—O4C	1.9679 (19)	C8A—H81A	0.9700
Zn1—O2A	1.9686 (18)	C8A—H82A	0.9700
Zn1—O4D	1.9857 (18)	C8D—N3D	1.458 (4)
Zn1—O1A	2.1037 (17)	C8D—C9D	1.508 (4)
Zn1—O3D	2.1604 (18)	C8D—H81D	0.9700
Zn2—O4D	1.9586 (18)	C8D—H82D	0.9700
Zn2—O2B	1.9707 (19)	C8C—N1C	1.453 (4)
Zn2—O4A	2.0111 (18)	C8C—C9C	1.528 (4)
Zn2—O1B	2.0945 (17)	C8C—H81C	0.9700
Zn2—O3A	2.1599 (18)	C8C—H82C	0.9700
Zn3—O4A	1.9556 (17)	C8B—N1B	1.455 (4)
Zn3—O2C	1.9877 (17)	C8B—C9B	1.513 (4)
Zn3—O4B	1.9930 (18)	C8B—H81B	0.9700
Zn3—O1C	2.0982 (18)	C8B—H82B	0.9700
Zn3—O3B	2.1476 (18)	C9—N3	1.442 (6)
Zn4—O1D	1.9618 (19)	C9—H9E	0.9600
Zn4—O4B	1.9712 (17)	C9—H9F	0.9600
Zn4—O4C	1.9832 (17)	C9—H9G	0.9600
Zn4—O2D	2.0947 (18)	C9B—N2B	1.464 (4)
Zn4—O3C	2.1553 (18)	C9B—H91B	0.9700
Zn5—O1A	2.0026 (18)	C9B—H92B	0.9700
Zn5—O3A	2.0090 (17)	C9A—N2A	1.467 (3)
Zn5—O5W	2.033 (2)	C9A—H91A	0.9700
Zn5—N1A	2.082 (2)	C9A—H92A	0.9700
Zn5—N2A	2.094 (2)	C9C—N2C	1.462 (3)
Zn6—O1B	1.9821 (17)	C9C—H91C	0.9700
Zn6—O3B	2.0121 (18)	C9C—H92C	0.9700
Zn6—O6W	2.0468 (18)	C9D—N4D	1.463 (3)
Zn6—N2B	2.066 (2)	C9D—H91D	0.9700
Zn6—N1B	2.076 (2)	C9D—H92D	0.9700
Zn7—O1C	1.9892 (17)	C10—O4	1.238 (4)
Zn7—O3C	2.0084 (17)	C10—N4	1.315 (4)
Zn7—O7W	2.029 (2)	C10—H10	0.9300
Zn7—N2C	2.055 (2)	C10D—N4D	1.276 (4)
Zn7—N1C	2.102 (2)	C10D—C11D	1.448 (4)
Zn8—O2D	2.0145 (19)	C10D—H10D	0.9300
Zn8—O3D	2.0233 (18)	C10C—N2C	1.278 (3)
Zn8—O8W	2.0341 (18)	C10C—C11C	1.434 (4)
Zn8—N3D	2.070 (2)	C10C—H10C	0.9300
Zn8—N4D	2.072 (2)	C10A—N2A	1.266 (4)
C1—O1	1.195 (4)	C10A—C11A	1.451 (4)
C1—N1	1.319 (4)	C10A—H10A	0.9300
C1—H1	0.9300	C10B—N2B	1.270 (4)
C1C—C6C	1.409 (4)	C10B—C11B	1.441 (4)
C1C—C2C	1.414 (3)	C10B—H10B	0.9300
C1C—C7C	1.441 (4)	C11—N4	1.454 (4)
C1B—C2B	1.404 (4)	C11—H11E	0.9600
C1B—C6B	1.416 (4)	C11—H11F	0.9600
C1B—C7B	1.448 (4)	C11—H11G	0.9600
C1D—O1D	1.334 (3)	C11A—C16A	1.404 (4)
C1D—C2D	1.389 (4)	C11A—C12A	1.408 (4)
C1D—C6D	1.429 (4)	C11B—C12B	1.408 (4)
C1A—C6A	1.411 (4)	C11B—C16B	1.414 (4)
C1A—C2A	1.419 (4)	C11D—C12D	1.402 (4)
C1A—C7A	1.433 (4)	C11D—C16D	1.412 (4)
C2—N1	1.450 (6)	C11C—C16C	1.411 (4)
C2—H2E	0.9600	C11C—C12C	1.414 (4)
C2—H2F	0.9600	C12—N4	1.432 (4)
C2—H2G	0.9600	C12—H12E	0.9600
C2C—C3C	1.376 (4)	C12—H12F	0.9600
C2C—H2C	0.9300	C12—H12G	0.9600
C2D—C3D	1.390 (4)	C12B—C13B	1.364 (4)
C2D—H2D	0.9300	C12B—H12B	0.9300
C2B—C3B	1.370 (4)	C12D—C13D	1.371 (4)
C2B—H2B	0.9300	C12D—H12D	0.9300
C2A—C3A	1.356 (5)	C12A—C13A	1.376 (4)
C2A—H2A	0.9300	C12A—H12A	0.9300
C3—N1	1.434 (5)	C12C—C13C	1.369 (4)
C3—H3E	0.9600	C12C—H12C	0.9300
C3—H3F	0.9600	C13—O5	1.235 (4)
C3—H3G	0.9600	C13—N5	1.322 (4)
C3C—C4C	1.396 (4)	C13—H13	0.9300
C3C—H3C	0.9300	C13A—C14A	1.395 (4)
C3B—C4B	1.388 (4)	C13A—H13A	0.9300
C3B—H3B	0.9300	C13C—C14C	1.395 (4)
C3D—C4D	1.357 (5)	C13C—H13C	0.9300
C3D—H3D	0.9300	C13B—C14B	1.387 (4)
C3A—C4A	1.405 (4)	C13B—H13B	0.9300
C3A—H3A	0.9300	C13D—C14D	1.389 (4)
C4—O2	1.237 (3)	C13D—H13D	0.9300
C4—N2	1.319 (4)	C14—N5	1.456 (4)
C4—H4	0.9300	C14—H14E	0.9600
C4C—C5C	1.389 (3)	C14—H14F	0.9600
C4C—H4C	0.9300	C14—H14G	0.9600
C4A—C5A	1.390 (4)	C14D—C15D	1.373 (4)
C4A—H4A	0.9300	C14D—H14D	0.9300
C4B—C5B	1.384 (4)	C14B—C15B	1.385 (4)
C4B—H4B	0.9300	C14B—H14B	0.9300
C4D—C5D	1.419 (4)	C14A—C15A	1.376 (4)
C4D—H4D	0.9300	C14A—H14A	0.9300
C5—N2	1.450 (4)	C14C—C15C	1.382 (4)
C5—H5E	0.9600	C14C—H14C	0.9300
C5—H5F	0.9600	C15—N5	1.439 (4)
C5—H5G	0.9600	C15—H15E	0.9600
C5B—O2B	1.340 (3)	C15—H15F	0.9600
C5B—C6B	1.420 (4)	C15—H15G	0.9600
C5C—O2C	1.326 (3)	C15D—O4D	1.358 (3)
C5C—C6C	1.431 (4)	C15D—C16D	1.417 (4)
C5A—O2A	1.338 (3)	C15A—O4A	1.354 (3)
C5A—C6A	1.412 (4)	C15A—C16A	1.433 (4)
C5D—C6D	1.403 (4)	C15C—O4C	1.365 (3)
C5D—C7D	1.442 (4)	C15C—C16C	1.419 (4)
C6—N2	1.440 (4)	C15B—O4B	1.352 (3)
C6—H6E	0.9600	C15B—C16B	1.423 (4)
C6—H6F	0.9600	C16D—O3D	1.336 (3)
C6—H6G	0.9600	C16A—O3A	1.329 (3)
C6C—O1C	1.334 (3)	C16B—O3B	1.327 (3)
C6D—O2D	1.333 (3)	C16C—O3C	1.338 (3)
C6B—O1B	1.344 (3)	C17—N6	1.458 (5)
C6A—O1A	1.348 (3)	C17—H17E	0.9600
C7—O3	1.288 (9)	C17—H17F	0.9600
C7—N3	1.294 (7)	C17—H17G	0.9600
C7—H7	0.9300	C18—N6	1.439 (4)
C7A—N1A	1.263 (4)	C18—H18E	0.9600
C7A—H7A	0.9300	C18—H18F	0.9600
C7C—N1C	1.274 (3)	C18—H18G	0.9600
C7C—H7C	0.9300	C19—O6	1.230 (4)
C7B—N1B	1.267 (4)	C19—N6	1.343 (4)
C7B—H7B	0.9300	C19—H19	0.9300
C7D—N3D	1.275 (4)	O8W—H81W	0.8500
C7D—H7D	0.9300	O8W—H82W	0.8500
C8—N3	1.453 (7)	O7W—H21W	0.8500
C8—H8E	0.9600	O7W—H22W	0.8500
C8—H8F	0.9600	O6W—H61W	0.8499
C8—H8G	0.9600	O6W—H62W	0.8500
C8A—N1A	1.458 (4)	O5W—H51W	0.8500
C8A—C9A	1.518 (4)	O5W—H52W	0.8499
			
O4C—Zn1—O2A	108.71 (8)	N2A—C9A—H91A	109.9
O4C—Zn1—O4D	127.05 (7)	C8A—C9A—H91A	109.9
O2A—Zn1—O4D	123.80 (8)	N2A—C9A—H92A	109.9
O4C—Zn1—O1A	101.78 (7)	C8A—C9A—H92A	109.9
O2A—Zn1—O1A	80.34 (7)	H91A—C9A—H92A	108.3
O4D—Zn1—O1A	93.61 (7)	N2C—C9C—C8C	108.1 (2)
O4C—Zn1—O3D	96.81 (7)	N2C—C9C—H91C	110.1
O2A—Zn1—O3D	89.28 (7)	C8C—C9C—H91C	110.1
O4D—Zn1—O3D	78.74 (7)	N2C—C9C—H92C	110.1
O1A—Zn1—O3D	160.82 (7)	C8C—C9C—H92C	110.1
O4D—Zn2—O2B	116.57 (8)	H91C—C9C—H92C	108.4
O4D—Zn2—O4A	121.50 (7)	N4D—C9D—C8D	107.8 (2)
O2B—Zn2—O4A	120.81 (8)	N4D—C9D—H91D	110.1
O4D—Zn2—O1B	103.87 (7)	C8D—C9D—H91D	110.1
O2B—Zn2—O1B	81.01 (7)	N4D—C9D—H92D	110.1
O4A—Zn2—O1B	95.32 (7)	C8D—C9D—H92D	110.1
O4D—Zn2—O3A	93.54 (7)	H91D—C9D—H92D	108.5
O2B—Zn2—O3A	87.95 (7)	O4—C10—N4	124.4 (3)
O4A—Zn2—O3A	78.34 (7)	O4—C10—H10	117.8
O1B—Zn2—O3A	162.20 (7)	N4—C10—H10	117.8
O4A—Zn3—O2C	120.90 (7)	N4D—C10D—C11D	125.6 (3)
O4A—Zn3—O4B	120.99 (7)	N4D—C10D—H10D	117.2
O2C—Zn3—O4B	117.04 (7)	C11D—C10D—H10D	117.2
O4A—Zn3—O1C	104.98 (7)	N2C—C10C—C11C	126.2 (3)
O2C—Zn3—O1C	80.22 (7)	N2C—C10C—H10C	116.9
O4B—Zn3—O1C	94.43 (7)	C11C—C10C—H10C	116.9
O4A—Zn3—O3B	92.71 (7)	N2A—C10A—C11A	126.0 (3)
O2C—Zn3—O3B	87.42 (7)	N2A—C10A—H10A	117.0
O4B—Zn3—O3B	79.38 (7)	C11A—C10A—H10A	117.0
O1C—Zn3—O3B	161.88 (7)	N2B—C10B—C11B	125.9 (3)
O1D—Zn4—O4B	115.92 (7)	N2B—C10B—H10B	117.1
O1D—Zn4—O4C	119.28 (8)	C11B—C10B—H10B	117.1
O4B—Zn4—O4C	124.06 (8)	N4—C11—H11E	109.5
O1D—Zn4—O2D	81.38 (7)	N4—C11—H11F	109.5
O4B—Zn4—O2D	100.78 (7)	H11E—C11—H11F	109.5
O4C—Zn4—O2D	95.61 (7)	N4—C11—H11G	109.5
O1D—Zn4—O3C	89.89 (8)	H11E—C11—H11G	109.5
O4B—Zn4—O3C	92.67 (7)	H11F—C11—H11G	109.5
O4C—Zn4—O3C	79.27 (7)	C16A—C11A—C12A	119.9 (3)
O2D—Zn4—O3C	166.16 (7)	C16A—C11A—C10A	123.7 (3)
O1A—Zn5—O3A	97.27 (7)	C12A—C11A—C10A	116.3 (3)
O1A—Zn5—O5W	101.29 (8)	C12B—C11B—C16B	119.6 (3)
O3A—Zn5—O5W	94.93 (7)	C12B—C11B—C10B	116.8 (3)
O1A—Zn5—N1A	88.62 (8)	C16B—C11B—C10B	123.5 (3)
O3A—Zn5—N1A	165.08 (9)	C12D—C11D—C16D	119.9 (3)
O5W—Zn5—N1A	97.34 (8)	C12D—C11D—C10D	115.7 (3)
O1A—Zn5—N2A	146.57 (9)	C16D—C11D—C10D	124.4 (3)
O3A—Zn5—N2A	88.97 (8)	C16C—C11C—C12C	119.7 (3)
O5W—Zn5—N2A	110.88 (9)	C16C—C11C—C10C	124.1 (2)
N1A—Zn5—N2A	78.76 (9)	C12C—C11C—C10C	116.1 (3)
O1B—Zn6—O3B	98.12 (7)	N4—C12—H12E	109.5
O1B—Zn6—O6W	104.09 (8)	N4—C12—H12F	109.5
O3B—Zn6—O6W	96.03 (7)	H12E—C12—H12F	109.5
O1B—Zn6—N2B	138.82 (8)	N4—C12—H12G	109.5
O3B—Zn6—N2B	88.98 (9)	H12E—C12—H12G	109.5
O6W—Zn6—N2B	115.48 (8)	H12F—C12—H12G	109.5
O1B—Zn6—N1B	88.81 (8)	C13B—C12B—C11B	121.6 (3)
O3B—Zn6—N1B	168.17 (9)	C13B—C12B—H12B	119.2
O6W—Zn6—N1B	91.52 (9)	C11B—C12B—H12B	119.2
N2B—Zn6—N1B	79.55 (10)	C13D—C12D—C11D	121.4 (3)
O1C—Zn7—O3C	97.35 (7)	C13D—C12D—H12D	119.3
O1C—Zn7—O7W	107.21 (8)	C11D—C12D—H12D	119.3
O3C—Zn7—O7W	94.96 (7)	C13A—C12A—C11A	121.6 (3)
O1C—Zn7—N2C	135.42 (9)	C13A—C12A—H12A	119.2
O3C—Zn7—N2C	90.35 (8)	C11A—C12A—H12A	119.2
O7W—Zn7—N2C	115.80 (9)	C13C—C12C—C11C	121.2 (3)
O1C—Zn7—N1C	87.35 (8)	C13C—C12C—H12C	119.4
O3C—Zn7—N1C	168.75 (8)	C11C—C12C—H12C	119.4
O7W—Zn7—N1C	93.39 (9)	O5—C13—N5	125.2 (3)
N2C—Zn7—N1C	79.22 (9)	O5—C13—H13	117.4
O2D—Zn8—O3D	97.69 (7)	N5—C13—H13	117.4
O2D—Zn8—O8W	107.45 (8)	C12A—C13A—C14A	118.8 (3)
O3D—Zn8—O8W	94.48 (7)	C12A—C13A—H13A	120.6
O2D—Zn8—N3D	88.62 (9)	C14A—C13A—H13A	120.6
O3D—Zn8—N3D	168.03 (8)	C12C—C13C—C14C	119.3 (3)
O8W—Zn8—N3D	93.31 (8)	C12C—C13C—H13C	120.4
O2D—Zn8—N4D	142.54 (8)	C14C—C13C—H13C	120.4
O3D—Zn8—N4D	89.95 (8)	C12B—C13B—C14B	119.1 (3)
O8W—Zn8—N4D	108.43 (8)	C12B—C13B—H13B	120.4
N3D—Zn8—N4D	78.95 (9)	C14B—C13B—H13B	120.4
O1—C1—N1	125.4 (4)	C12D—C13D—C14D	119.0 (3)
O1—C1—H1	117.3	C12D—C13D—H13D	120.5
N1—C1—H1	117.3	C14D—C13D—H13D	120.5
C6C—C1C—C2C	119.6 (2)	N5—C14—H14E	109.5
C6C—C1C—C7C	123.1 (2)	N5—C14—H14F	109.5
C2C—C1C—C7C	116.9 (2)	H14E—C14—H14F	109.5
C2B—C1B—C6B	119.7 (3)	N5—C14—H14G	109.5
C2B—C1B—C7B	116.8 (3)	H14E—C14—H14G	109.5
C6B—C1B—C7B	123.5 (3)	H14F—C14—H14G	109.5
O1D—C1D—C2D	122.1 (3)	C15D—C14D—C13D	121.3 (3)
O1D—C1D—C6D	118.2 (2)	C15D—C14D—H14D	119.3
C2D—C1D—C6D	119.7 (3)	C13D—C14D—H14D	119.3
C6A—C1A—C2A	119.2 (3)	C15B—C14B—C13B	121.6 (3)
C6A—C1A—C7A	123.9 (3)	C15B—C14B—H14B	119.2
C2A—C1A—C7A	116.8 (3)	C13B—C14B—H14B	119.2
N1—C2—H2E	109.5	C15A—C14A—C13A	121.4 (3)
N1—C2—H2F	109.5	C15A—C14A—H14A	119.3
H2E—C2—H2F	109.5	C13A—C14A—H14A	119.3
N1—C2—H2G	109.5	C15C—C14C—C13C	121.0 (3)
H2E—C2—H2G	109.5	C15C—C14C—H14C	119.5
H2F—C2—H2G	109.5	C13C—C14C—H14C	119.5
C3C—C2C—C1C	121.5 (3)	N5—C15—H15E	109.5
C3C—C2C—H2C	119.3	N5—C15—H15F	109.5
C1C—C2C—H2C	119.3	H15E—C15—H15F	109.5
C1D—C2D—C3D	121.3 (3)	N5—C15—H15G	109.5
C1D—C2D—H2D	119.4	H15E—C15—H15G	109.5
C3D—C2D—H2D	119.4	H15F—C15—H15G	109.5
C3B—C2B—C1B	120.9 (3)	O4D—C15D—C14D	122.3 (2)
C3B—C2B—H2B	119.6	O4D—C15D—C16D	117.0 (2)
C1B—C2B—H2B	119.6	C14D—C15D—C16D	120.8 (3)
C3A—C2A—C1A	121.1 (3)	O4A—C15A—C14A	122.4 (2)
C3A—C2A—H2A	119.4	O4A—C15A—C16A	117.1 (2)
C1A—C2A—H2A	119.4	C14A—C15A—C16A	120.4 (3)
N1—C3—H3E	109.5	O4C—C15C—C14C	121.9 (2)
N1—C3—H3F	109.5	O4C—C15C—C16C	117.4 (2)
H3E—C3—H3F	109.5	C14C—C15C—C16C	120.5 (3)
N1—C3—H3G	109.5	O4B—C15B—C14B	122.2 (3)
H3E—C3—H3G	109.5	O4B—C15B—C16B	117.8 (2)
H3F—C3—H3G	109.5	C14B—C15B—C16B	119.9 (3)
C2C—C3C—C4C	119.1 (2)	O3D—C16D—C11D	125.7 (2)
C2C—C3C—H3C	120.5	O3D—C16D—C15D	116.7 (2)
C4C—C3C—H3C	120.5	C11D—C16D—C15D	117.6 (3)
C2B—C3B—C4B	119.7 (3)	O3A—C16A—C11A	125.9 (2)
C2B—C3B—H3B	120.2	O3A—C16A—C15A	116.2 (2)
C4B—C3B—H3B	120.2	C11A—C16A—C15A	117.8 (2)
C4D—C3D—C2D	119.4 (3)	O3B—C16B—C11B	125.4 (3)
C4D—C3D—H3D	120.3	O3B—C16B—C15B	116.6 (2)
C2D—C3D—H3D	120.3	C11B—C16B—C15B	118.0 (2)
C2A—C3A—C4A	119.9 (3)	O3C—C16C—C11C	125.7 (2)
C2A—C3A—H3A	120.1	O3C—C16C—C15C	116.4 (2)
C4A—C3A—H3A	120.1	C11C—C16C—C15C	117.9 (2)
O2—C4—N2	125.6 (3)	N6—C17—H17E	109.5
O2—C4—H4	117.2	N6—C17—H17F	109.5
N2—C4—H4	117.2	H17E—C17—H17F	109.5
C5C—C4C—C3C	121.6 (3)	N6—C17—H17G	109.5
C5C—C4C—H4C	119.2	H17E—C17—H17G	109.5
C3C—C4C—H4C	119.2	H17F—C17—H17G	109.5
C5A—C4A—C3A	120.8 (3)	N6—C18—H18E	109.5
C5A—C4A—H4A	119.6	N6—C18—H18F	109.5
C3A—C4A—H4A	119.6	H18E—C18—H18F	109.5
C5B—C4B—C3B	121.7 (3)	N6—C18—H18G	109.5
C5B—C4B—H4B	119.1	H18E—C18—H18G	109.5
C3B—C4B—H4B	119.1	H18F—C18—H18G	109.5
C3D—C4D—C5D	121.8 (3)	O6—C19—N6	123.3 (3)
C3D—C4D—H4D	119.1	O6—C19—H19	118.4
C5D—C4D—H4D	119.1	N6—C19—H19	118.4
N2—C5—H5E	109.5	Zn8—O8W—H81W	109.6
N2—C5—H5F	109.5	Zn8—O8W—H82W	109.3
H5E—C5—H5F	109.5	H81W—O8W—H82W	109.8
N2—C5—H5G	109.5	C1D—O1D—Zn4	112.90 (17)
H5E—C5—H5G	109.5	C6B—O1B—Zn6	127.45 (15)
H5F—C5—H5G	109.5	C6B—O1B—Zn2	110.90 (15)
O2B—C5B—C4B	122.5 (3)	Zn6—O1B—Zn2	121.33 (9)
O2B—C5B—C6B	118.2 (2)	C6A—O1A—Zn5	128.11 (17)
C4B—C5B—C6B	119.3 (3)	C6A—O1A—Zn1	109.46 (16)
O2C—C5C—C4C	122.6 (2)	Zn5—O1A—Zn1	120.59 (8)
O2C—C5C—C6C	117.8 (2)	C6C—O1C—Zn7	128.69 (16)
C4C—C5C—C6C	119.6 (2)	C6C—O1C—Zn3	111.46 (15)
O2A—C5A—C4A	122.2 (3)	Zn7—O1C—Zn3	119.81 (8)
O2A—C5A—C6A	118.1 (2)	Zn7—O7W—H21W	109.6
C4A—C5A—C6A	119.7 (3)	Zn7—O7W—H22W	109.3
C6D—C5D—C4D	119.3 (3)	H21W—O7W—H22W	109.8
C6D—C5D—C7D	124.2 (3)	C5A—O2A—Zn1	112.43 (18)
C4D—C5D—C7D	116.4 (3)	C6D—O2D—Zn8	128.15 (17)
N2—C6—H6E	109.5	C6D—O2D—Zn4	109.64 (16)
N2—C6—H6F	109.5	Zn8—O2D—Zn4	121.49 (9)
H6E—C6—H6F	109.5	C5B—O2B—Zn2	113.89 (16)
N2—C6—H6G	109.5	C5C—O2C—Zn3	114.44 (16)
H6E—C6—H6G	109.5	C16D—O3D—Zn8	126.48 (16)
H6F—C6—H6G	109.5	C16D—O3D—Zn1	106.15 (15)
O1C—C6C—C1C	125.5 (2)	Zn8—O3D—Zn1	120.73 (8)
O1C—C6C—C5C	115.9 (2)	Zn6—O6W—H61W	109.6
C1C—C6C—C5C	118.6 (2)	Zn6—O6W—H62W	109.3
O2D—C6D—C5D	125.3 (3)	H61W—O6W—H62W	109.8
O2D—C6D—C1D	116.2 (2)	C16B—O3B—Zn6	127.70 (17)
C5D—C6D—C1D	118.4 (3)	C16B—O3B—Zn3	105.16 (16)
O1B—C6B—C1B	125.4 (2)	Zn6—O3B—Zn3	119.12 (9)
O1B—C6B—C5B	115.9 (2)	C16C—O3C—Zn7	125.95 (17)
C1B—C6B—C5B	118.7 (3)	C16C—O3C—Zn4	106.01 (15)
O1A—C6A—C1A	125.1 (3)	Zn7—O3C—Zn4	120.79 (8)
O1A—C6A—C5A	115.7 (2)	C16A—O3A—Zn5	128.08 (16)
C1A—C6A—C5A	119.2 (2)	C16A—O3A—Zn2	104.78 (14)
O3—C7—N3	119.7 (9)	Zn5—O3A—Zn2	120.08 (9)
O3—C7—H7	120.1	C15D—O4D—Zn2	119.59 (15)
N3—C7—H7	120.1	C15D—O4D—Zn1	110.17 (15)
N1A—C7A—C1A	126.2 (2)	Zn2—O4D—Zn1	122.28 (9)
N1A—C7A—H7A	116.9	Zn5—O5W—H51W	109.5
C1A—C7A—H7A	116.9	Zn5—O5W—H52W	109.4
N1C—C7C—C1C	124.9 (3)	H51W—O5W—H52W	109.8
N1C—C7C—H7C	117.6	C15B—O4B—Zn4	122.26 (15)
C1C—C7C—H7C	117.6	C15B—O4B—Zn3	108.25 (15)
N1B—C7B—C1B	124.7 (3)	Zn4—O4B—Zn3	122.88 (9)
N1B—C7B—H7B	117.7	C15C—O4C—Zn1	121.56 (16)
C1B—C7B—H7B	117.7	C15C—O4C—Zn4	109.25 (16)
N3D—C7D—C5D	125.2 (3)	Zn1—O4C—Zn4	121.66 (9)
N3D—C7D—H7D	117.4	C15A—O4A—Zn3	122.26 (17)
C5D—C7D—H7D	117.4	C15A—O4A—Zn2	108.10 (15)
N3—C8—H8E	109.5	Zn3—O4A—Zn2	126.44 (9)
N3—C8—H8F	109.5	C1—N1—C3	123.2 (4)
H8E—C8—H8F	109.5	C1—N1—C2	120.0 (3)
N3—C8—H8G	109.5	C3—N1—C2	116.8 (4)
H8E—C8—H8G	109.5	C7B—N1B—C8B	122.0 (3)
H8F—C8—H8G	109.5	C7B—N1B—Zn6	128.1 (2)
N1A—C8A—C9A	107.2 (2)	C8B—N1B—Zn6	109.83 (19)
N1A—C8A—H81A	110.3	C7A—N1A—C8A	121.5 (2)
C9A—C8A—H81A	110.3	C7A—N1A—Zn5	127.8 (2)
N1A—C8A—H82A	110.3	C8A—N1A—Zn5	110.20 (17)
C9A—C8A—H82A	110.3	C7C—N1C—C8C	121.0 (2)
H81A—C8A—H82A	108.5	C7C—N1C—Zn7	127.8 (2)
N3D—C8D—C9D	107.2 (2)	C8C—N1C—Zn7	110.97 (17)
N3D—C8D—H81D	110.3	C10B—N2B—C9B	118.8 (3)
C9D—C8D—H81D	110.3	C10B—N2B—Zn6	127.7 (2)
N3D—C8D—H82D	110.3	C9B—N2B—Zn6	113.38 (19)
C9D—C8D—H82D	110.3	C10A—N2A—C9A	118.3 (3)
H81D—C8D—H82D	108.5	C10A—N2A—Zn5	127.1 (2)
N1C—C8C—C9C	106.4 (2)	C9A—N2A—Zn5	114.19 (19)
N1C—C8C—H81C	110.5	C10C—N2C—C9C	119.1 (2)
C9C—C8C—H81C	110.5	C10C—N2C—Zn7	126.4 (2)
N1C—C8C—H82C	110.5	C9C—N2C—Zn7	114.42 (17)
C9C—C8C—H82C	110.5	C4—N2—C6	121.5 (2)
H81C—C8C—H82C	108.6	C4—N2—C5	122.8 (2)
N1B—C8B—C9B	106.4 (2)	C6—N2—C5	115.7 (2)
N1B—C8B—H81B	110.5	C7D—N3D—C8D	120.9 (3)
C9B—C8B—H81B	110.5	C7D—N3D—Zn8	128.3 (2)
N1B—C8B—H82B	110.5	C8D—N3D—Zn8	110.20 (19)
C9B—C8B—H82B	110.5	C7—N3—C9	122.5 (6)
H81B—C8B—H82B	108.6	C7—N3—C8	121.3 (7)
N3—C9—H9E	109.5	C9—N3—C8	116.2 (5)
N3—C9—H9F	109.5	C10—N4—C12	121.2 (3)
H9E—C9—H9F	109.5	C10—N4—C11	122.8 (3)
N3—C9—H9G	109.5	C12—N4—C11	115.6 (3)
H9E—C9—H9G	109.5	C10D—N4D—C9D	118.5 (2)
H9F—C9—H9G	109.5	C10D—N4D—Zn8	127.0 (2)
N2B—C9B—C8B	108.0 (3)	C9D—N4D—Zn8	114.52 (18)
N2B—C9B—H91B	110.1	C13—N5—C15	122.3 (3)
C8B—C9B—H91B	110.1	C13—N5—C14	119.9 (3)
N2B—C9B—H92B	110.1	C15—N5—C14	117.4 (3)
C8B—C9B—H92B	110.1	C19—N6—C18	121.2 (3)
H91B—C9B—H92B	108.4	C19—N6—C17	120.0 (3)
N2A—C9A—C8A	108.9 (2)	C18—N6—C17	118.3 (3)
			
C6C—C1C—C2C—C3C	−1.0 (4)	O4C—Zn1—O3D—Zn8	−53.03 (10)
C7C—C1C—C2C—C3C	171.6 (3)	O2A—Zn1—O3D—Zn8	55.73 (11)
O1D—C1D—C2D—C3D	178.0 (3)	O4D—Zn1—O3D—Zn8	−179.54 (11)
C6D—C1D—C2D—C3D	−3.3 (4)	O1A—Zn1—O3D—Zn8	112.61 (19)
C6B—C1B—C2B—C3B	0.2 (5)	C11B—C16B—O3B—Zn6	8.0 (3)
C7B—C1B—C2B—C3B	−176.7 (3)	C15B—C16B—O3B—Zn6	−172.14 (15)
C6A—C1A—C2A—C3A	0.3 (5)	C11B—C16B—O3B—Zn3	155.8 (2)
C7A—C1A—C2A—C3A	176.8 (3)	C15B—C16B—O3B—Zn3	−24.3 (2)
C1C—C2C—C3C—C4C	−0.8 (4)	O1B—Zn6—O3B—C16B	−147.45 (19)
C1B—C2B—C3B—C4B	1.1 (5)	O6W—Zn6—O3B—C16B	107.34 (19)
C1D—C2D—C3D—C4D	0.8 (5)	N2B—Zn6—O3B—C16B	−8.17 (19)
C1A—C2A—C3A—C4A	1.3 (5)	N1B—Zn6—O3B—C16B	−22.1 (5)
C2C—C3C—C4C—C5C	1.3 (4)	O1B—Zn6—O3B—Zn3	68.57 (10)
C2A—C3A—C4A—C5A	−0.2 (5)	O6W—Zn6—O3B—Zn3	−36.63 (10)
C2B—C3B—C4B—C5B	−0.1 (5)	N2B—Zn6—O3B—Zn3	−152.15 (10)
C2D—C3D—C4D—C5D	1.3 (5)	N1B—Zn6—O3B—Zn3	−166.0 (3)
C3B—C4B—C5B—O2B	179.6 (3)	O4A—Zn3—O3B—C16B	150.77 (14)
C3B—C4B—C5B—C6B	−2.1 (5)	O2C—Zn3—O3B—C16B	−88.40 (15)
C3C—C4C—C5C—O2C	179.0 (3)	O4B—Zn3—O3B—C16B	29.73 (14)
C3C—C4C—C5C—C6C	0.1 (4)	O1C—Zn3—O3B—C16B	−41.6 (3)
C3A—C4A—C5A—O2A	178.8 (3)	O4A—Zn3—O3B—Zn6	−58.05 (9)
C3A—C4A—C5A—C6A	−2.5 (5)	O2C—Zn3—O3B—Zn6	62.78 (10)
C3D—C4D—C5D—C6D	−0.8 (5)	O4B—Zn3—O3B—Zn6	−179.09 (10)
C3D—C4D—C5D—C7D	−178.2 (3)	O1C—Zn3—O3B—Zn6	109.6 (2)
C2C—C1C—C6C—O1C	−177.5 (2)	C11C—C16C—O3C—Zn7	−8.6 (4)
C7C—C1C—C6C—O1C	10.4 (4)	C15C—C16C—O3C—Zn7	171.60 (17)
C2C—C1C—C6C—C5C	2.3 (4)	C11C—C16C—O3C—Zn4	−158.6 (2)
C7C—C1C—C6C—C5C	−169.9 (3)	C15C—C16C—O3C—Zn4	21.6 (3)
O2C—C5C—C6C—O1C	−1.1 (3)	O1C—Zn7—O3C—C16C	147.7 (2)
C4C—C5C—C6C—O1C	177.9 (2)	O7W—Zn7—O3C—C16C	−104.2 (2)
O2C—C5C—C6C—C1C	179.2 (2)	N2C—Zn7—O3C—C16C	11.7 (2)
C4C—C5C—C6C—C1C	−1.8 (4)	N1C—Zn7—O3C—C16C	33.6 (5)
C4D—C5D—C6D—O2D	−179.5 (3)	O1C—Zn7—O3C—Zn4	−66.33 (10)
C7D—C5D—C6D—O2D	−2.4 (4)	O7W—Zn7—O3C—Zn4	41.77 (11)
C4D—C5D—C6D—C1D	−1.6 (4)	N2C—Zn7—O3C—Zn4	157.70 (11)
C7D—C5D—C6D—C1D	175.5 (3)	O1D—Zn4—O3C—C16C	92.03 (16)
O1D—C1D—C6D—O2D	0.5 (4)	O4B—Zn4—O3C—C16C	−152.03 (16)
C2D—C1D—C6D—O2D	−178.3 (2)	O4C—Zn4—O3C—C16C	−27.85 (16)
O1D—C1D—C6D—C5D	−177.6 (2)	O2D—Zn4—O3C—C16C	41.4 (4)
C2D—C1D—C6D—C5D	3.6 (4)	O1D—Zn4—O3C—Zn7	−59.84 (10)
C2B—C1B—C6B—O1B	177.4 (3)	O4B—Zn4—O3C—Zn7	56.11 (10)
C7B—C1B—C6B—O1B	−5.9 (5)	O4C—Zn4—O3C—Zn7	−179.71 (11)
C2B—C1B—C6B—C5B	−2.4 (4)	O2D—Zn4—O3C—Zn7	−110.4 (3)
C7B—C1B—C6B—C5B	174.3 (3)	C11A—C16A—O3A—Zn5	−0.4 (4)
O2B—C5B—C6B—O1B	1.8 (4)	C15A—C16A—O3A—Zn5	177.49 (17)
C4B—C5B—C6B—O1B	−176.5 (3)	C11A—C16A—O3A—Zn2	−150.3 (2)
O2B—C5B—C6B—C1B	−178.3 (3)	C15A—C16A—O3A—Zn2	27.6 (3)
C4B—C5B—C6B—C1B	3.4 (4)	O1A—Zn5—O3A—C16A	147.6 (2)
C2A—C1A—C6A—O1A	175.5 (3)	O5W—Zn5—O3A—C16A	−110.3 (2)
C7A—C1A—C6A—O1A	−0.8 (5)	N1A—Zn5—O3A—C16A	35.0 (4)
C2A—C1A—C6A—C5A	−3.0 (4)	N2A—Zn5—O3A—C16A	0.6 (2)
C7A—C1A—C6A—C5A	−179.3 (3)	O1A—Zn5—O3A—Zn2	−66.45 (10)
O2A—C5A—C6A—O1A	4.2 (4)	O5W—Zn5—O3A—Zn2	35.62 (11)
C4A—C5A—C6A—O1A	−174.5 (3)	N1A—Zn5—O3A—Zn2	−179.1 (3)
O2A—C5A—C6A—C1A	−177.2 (3)	N2A—Zn5—O3A—Zn2	146.49 (11)
C4A—C5A—C6A—C1A	4.1 (5)	O4D—Zn2—O3A—C16A	−154.26 (16)
C6A—C1A—C7A—N1A	0.3 (5)	O2B—Zn2—O3A—C16A	89.24 (16)
C2A—C1A—C7A—N1A	−176.1 (3)	O4A—Zn2—O3A—C16A	−32.82 (16)
C6C—C1C—C7C—N1C	−9.6 (4)	O1B—Zn2—O3A—C16A	37.7 (3)
C2C—C1C—C7C—N1C	178.0 (3)	O4D—Zn2—O3A—Zn5	52.88 (10)
C2B—C1B—C7B—N1B	−174.0 (3)	O2B—Zn2—O3A—Zn5	−63.62 (10)
C6B—C1B—C7B—N1B	9.1 (5)	O4A—Zn2—O3A—Zn5	174.32 (11)
C6D—C5D—C7D—N3D	5.1 (5)	O1B—Zn2—O3A—Zn5	−115.1 (2)
C4D—C5D—C7D—N3D	−177.6 (3)	C14D—C15D—O4D—Zn2	58.3 (3)
N1B—C8B—C9B—N2B	49.8 (3)	C16D—C15D—O4D—Zn2	−123.3 (2)
N1A—C8A—C9A—N2A	−45.1 (3)	C14D—C15D—O4D—Zn1	−152.1 (2)
N1C—C8C—C9C—N2C	−48.4 (3)	C16D—C15D—O4D—Zn1	26.3 (3)
N3D—C8D—C9D—N4D	47.0 (3)	O2B—Zn2—O4D—C15D	−95.07 (19)
N2A—C10A—C11A—C16A	5.4 (5)	O4A—Zn2—O4D—C15D	96.95 (19)
N2A—C10A—C11A—C12A	−175.7 (3)	O1B—Zn2—O4D—C15D	−8.3 (2)
N2B—C10B—C11B—C12B	172.5 (2)	O3A—Zn2—O4D—C15D	175.44 (18)
N2B—C10B—C11B—C16B	−7.1 (4)	O2B—Zn2—O4D—Zn1	119.07 (10)
N4D—C10D—C11D—C12D	173.3 (3)	O4A—Zn2—O4D—Zn1	−48.90 (13)
N4D—C10D—C11D—C16D	−7.0 (4)	O1B—Zn2—O4D—Zn1	−154.17 (9)
N2C—C10C—C11C—C16C	7.1 (5)	O3A—Zn2—O4D—Zn1	29.59 (11)
N2C—C10C—C11C—C12C	−173.5 (3)	O4C—Zn1—O4D—C15D	−118.52 (16)
C16B—C11B—C12B—C13B	1.5 (4)	O2A—Zn1—O4D—C15D	52.99 (18)
C10B—C11B—C12B—C13B	−178.2 (2)	O1A—Zn1—O4D—C15D	133.78 (16)
C16D—C11D—C12D—C13D	1.1 (4)	O3D—Zn1—O4D—C15D	−28.46 (16)
C10D—C11D—C12D—C13D	−179.2 (2)	O4C—Zn1—O4D—Zn2	30.14 (14)
C16A—C11A—C12A—C13A	−1.3 (5)	O2A—Zn1—O4D—Zn2	−158.34 (9)
C10A—C11A—C12A—C13A	179.7 (3)	O1A—Zn1—O4D—Zn2	−77.55 (10)
C16C—C11C—C12C—C13C	−1.0 (4)	O3D—Zn1—O4D—Zn2	120.21 (11)
C10C—C11C—C12C—C13C	179.6 (3)	C14B—C15B—O4B—Zn4	57.7 (3)
C11A—C12A—C13A—C14A	0.8 (5)	C16B—C15B—O4B—Zn4	−125.7 (2)
C11C—C12C—C13C—C14C	1.6 (4)	C14B—C15B—O4B—Zn3	−150.1 (2)
C11B—C12B—C13B—C14B	−2.0 (4)	C16B—C15B—O4B—Zn3	26.4 (3)
C11D—C12D—C13D—C14D	−2.2 (4)	O1D—Zn4—O4B—C15B	−95.7 (2)
C12D—C13D—C14D—C15D	0.6 (4)	O4C—Zn4—O4B—C15B	94.2 (2)
C12B—C13B—C14B—C15B	−0.2 (4)	O2D—Zn4—O4B—C15B	−10.2 (2)
C12A—C13A—C14A—C15A	0.1 (4)	O3C—Zn4—O4B—C15B	173.08 (19)
C12C—C13C—C14C—C15C	1.4 (4)	O1D—Zn4—O4B—Zn3	116.14 (11)
C13D—C14D—C15D—O4D	−179.6 (2)	O4C—Zn4—O4B—Zn3	−53.88 (14)
C13D—C14D—C15D—C16D	2.1 (4)	O2D—Zn4—O4B—Zn3	−158.28 (10)
C13A—C14A—C15A—O4A	−176.7 (3)	O3C—Zn4—O4B—Zn3	24.97 (11)
C13A—C14A—C15A—C16A	−0.5 (4)	O4A—Zn3—O4B—C15B	−116.44 (16)
C13C—C14C—C15C—O4C	178.5 (3)	O2C—Zn3—O4B—C15B	51.85 (17)
C13C—C14C—C15C—C16C	−4.9 (4)	O1C—Zn3—O4B—C15B	133.13 (15)
C13B—C14B—C15B—O4B	179.3 (2)	O3B—Zn3—O4B—C15B	−29.68 (15)
C13B—C14B—C15B—C16B	2.8 (4)	O4A—Zn3—O4B—Zn4	35.51 (14)
C12D—C11D—C16D—O3D	−178.5 (2)	O2C—Zn3—O4B—Zn4	−156.20 (9)
C10D—C11D—C16D—O3D	1.8 (4)	O1C—Zn3—O4B—Zn4	−74.92 (11)
C12D—C11D—C16D—C15D	1.5 (4)	O3B—Zn3—O4B—Zn4	122.27 (11)
C10D—C11D—C16D—C15D	−178.2 (2)	C14C—C15C—O4C—Zn1	−60.1 (3)
O4D—C15D—C16D—O3D	−1.5 (3)	C16C—C15C—O4C—Zn1	123.3 (2)
C14D—C15D—C16D—O3D	177.0 (2)	C14C—C15C—O4C—Zn4	149.8 (2)
O4D—C15D—C16D—C11D	178.5 (2)	C16C—C15C—O4C—Zn4	−26.8 (3)
C14D—C15D—C16D—C11D	−3.1 (4)	O2A—Zn1—O4C—C15C	98.31 (18)
C12A—C11A—C16A—O3A	178.7 (3)	O4D—Zn1—O4C—C15C	−89.13 (19)
C10A—C11A—C16A—O3A	−2.4 (5)	O1A—Zn1—O4C—C15C	14.64 (19)
C12A—C11A—C16A—C15A	0.9 (4)	O3D—Zn1—O4C—C15C	−170.14 (17)
C10A—C11A—C16A—C15A	179.8 (3)	O2A—Zn1—O4C—Zn4	−115.24 (11)
O4A—C15A—C16A—O3A	−1.6 (4)	O4D—Zn1—O4C—Zn4	57.32 (14)
C14A—C15A—C16A—O3A	−178.0 (2)	O1A—Zn1—O4C—Zn4	161.09 (10)
O4A—C15A—C16A—C11A	176.4 (2)	O3D—Zn1—O4C—Zn4	−23.68 (11)
C14A—C15A—C16A—C11A	0.0 (4)	O1D—Zn4—O4C—C15C	−54.91 (18)
C12B—C11B—C16B—O3B	−179.0 (2)	O4B—Zn4—O4C—C15C	114.79 (16)
C10B—C11B—C16B—O3B	0.7 (4)	O2D—Zn4—O4C—C15C	−138.16 (16)
C12B—C11B—C16B—C15B	1.1 (4)	O3C—Zn4—O4C—C15C	28.85 (15)
C10B—C11B—C16B—C15B	−179.2 (2)	O1D—Zn4—O4C—Zn1	155.00 (9)
O4B—C15B—C16B—O3B	0.2 (3)	O4B—Zn4—O4C—Zn1	−35.29 (14)
C14B—C15B—C16B—O3B	176.9 (2)	O2D—Zn4—O4C—Zn1	71.76 (11)
O4B—C15B—C16B—C11B	−179.9 (2)	O3C—Zn4—O4C—Zn1	−121.23 (11)
C14B—C15B—C16B—C11B	−3.2 (4)	C14A—C15A—O4A—Zn3	−50.7 (3)
C12C—C11C—C16C—O3C	177.8 (3)	C16A—C15A—O4A—Zn3	133.0 (2)
C10C—C11C—C16C—O3C	−2.8 (5)	C14A—C15A—O4A—Zn2	148.4 (2)
C12C—C11C—C16C—C15C	−2.4 (4)	C16A—C15A—O4A—Zn2	−27.9 (3)
C10C—C11C—C16C—C15C	176.9 (3)	O2C—Zn3—O4A—C15A	93.16 (19)
O4C—C15C—C16C—O3C	1.8 (4)	O4B—Zn3—O4A—C15A	−99.00 (18)
C14C—C15C—C16C—O3C	−174.9 (2)	O1C—Zn3—O4A—C15A	5.72 (19)
O4C—C15C—C16C—C11C	−178.0 (2)	O3B—Zn3—O4A—C15A	−178.23 (18)
C14C—C15C—C16C—C11C	5.4 (4)	O2C—Zn3—O4A—Zn2	−109.52 (11)
C2D—C1D—O1D—Zn4	−171.7 (2)	O4B—Zn3—O4A—Zn2	58.32 (14)
C6D—C1D—O1D—Zn4	9.6 (3)	O1C—Zn3—O4A—Zn2	163.04 (10)
O4B—Zn4—O1D—C1D	86.74 (17)	O3B—Zn3—O4A—Zn2	−20.91 (11)
O4C—Zn4—O1D—C1D	−102.74 (17)	O4D—Zn2—O4A—C15A	119.18 (16)
O2D—Zn4—O1D—C1D	−11.11 (16)	O2B—Zn2—O4A—C15A	−48.30 (18)
O3C—Zn4—O1D—C1D	179.66 (16)	O1B—Zn2—O4A—C15A	−130.99 (16)
C1B—C6B—O1B—Zn6	−8.8 (4)	O3A—Zn2—O4A—C15A	32.18 (15)
C5B—C6B—O1B—Zn6	171.01 (18)	O4D—Zn2—O4A—Zn3	−40.76 (14)
C1B—C6B—O1B—Zn2	177.7 (2)	O2B—Zn2—O4A—Zn3	151.77 (10)
C5B—C6B—O1B—Zn2	−2.5 (3)	O1B—Zn2—O4A—Zn3	69.08 (11)
O3B—Zn6—O1B—C6B	−175.4 (2)	O3A—Zn2—O4A—Zn3	−127.76 (12)
O6W—Zn6—O1B—C6B	−77.1 (2)	O1—C1—N1—C3	−179.0 (5)
N2B—Zn6—O1B—C6B	86.8 (2)	O1—C1—N1—C2	0.4 (7)
N1B—Zn6—O1B—C6B	14.2 (2)	C1B—C7B—N1B—C8B	−174.5 (3)
O3B—Zn6—O1B—Zn2	−2.56 (11)	C1B—C7B—N1B—Zn6	2.3 (5)
O6W—Zn6—O1B—Zn2	95.79 (11)	C9B—C8B—N1B—C7B	129.3 (3)
N2B—Zn6—O1B—Zn2	−100.36 (15)	C9B—C8B—N1B—Zn6	−48.0 (3)
N1B—Zn6—O1B—Zn2	−172.94 (12)	O1B—Zn6—N1B—C7B	−11.3 (3)
O4D—Zn2—O1B—C6B	−113.39 (17)	O3B—Zn6—N1B—C7B	−137.4 (4)
O2B—Zn2—O1B—C6B	1.91 (17)	O6W—Zn6—N1B—C7B	92.8 (3)
O4A—Zn2—O1B—C6B	122.32 (17)	N2B—Zn6—N1B—C7B	−151.6 (3)
O3A—Zn2—O1B—C6B	54.3 (3)	O1B—Zn6—N1B—C8B	165.8 (2)
O4D—Zn2—O1B—Zn6	72.67 (11)	O3B—Zn6—N1B—C8B	39.7 (5)
O2B—Zn2—O1B—Zn6	−172.03 (12)	O6W—Zn6—N1B—C8B	−90.1 (2)
O4A—Zn2—O1B—Zn6	−51.63 (11)	N2B—Zn6—N1B—C8B	25.5 (2)
O3A—Zn2—O1B—Zn6	−119.7 (2)	C1A—C7A—N1A—C8A	174.9 (3)
C1A—C6A—O1A—Zn5	−3.7 (4)	C1A—C7A—N1A—Zn5	4.4 (5)
C5A—C6A—O1A—Zn5	174.85 (19)	C9A—C8A—N1A—C7A	−123.6 (3)
C1A—C6A—O1A—Zn1	−168.1 (2)	C9A—C8A—N1A—Zn5	48.3 (3)
C5A—C6A—O1A—Zn1	10.5 (3)	O1A—Zn5—N1A—C7A	−6.2 (3)
O3A—Zn5—O1A—C6A	−160.5 (2)	O3A—Zn5—N1A—C7A	107.5 (4)
O5W—Zn5—O1A—C6A	103.0 (2)	O5W—Zn5—N1A—C7A	−107.4 (3)
N1A—Zn5—O1A—C6A	5.7 (2)	N2A—Zn5—N1A—C7A	142.7 (3)
N2A—Zn5—O1A—C6A	−61.3 (3)	O1A—Zn5—N1A—C8A	−177.5 (2)
O3A—Zn5—O1A—Zn1	2.34 (11)	O3A—Zn5—N1A—C8A	−63.9 (4)
O5W—Zn5—O1A—Zn1	−94.20 (10)	O5W—Zn5—N1A—C8A	81.2 (2)
N1A—Zn5—O1A—Zn1	168.59 (11)	N2A—Zn5—N1A—C8A	−28.7 (2)
N2A—Zn5—O1A—Zn1	101.57 (15)	C1C—C7C—N1C—C8C	168.0 (3)
O4C—Zn1—O1A—C6A	91.96 (17)	C1C—C7C—N1C—Zn7	−6.0 (4)
O2A—Zn1—O1A—C6A	−15.31 (17)	C9C—C8C—N1C—C7C	−130.4 (3)
O4D—Zn1—O1A—C6A	−139.00 (17)	C9C—C8C—N1C—Zn7	44.5 (3)
O3D—Zn1—O1A—C6A	−73.5 (3)	O1C—Zn7—N1C—C7C	14.7 (3)
O4C—Zn1—O1A—Zn5	−73.78 (11)	O3C—Zn7—N1C—C7C	129.7 (4)
O2A—Zn1—O1A—Zn5	178.94 (12)	O7W—Zn7—N1C—C7C	−92.4 (3)
O4D—Zn1—O1A—Zn5	55.25 (11)	N2C—Zn7—N1C—C7C	151.9 (3)
O3D—Zn1—O1A—Zn5	120.78 (18)	O1C—Zn7—N1C—C8C	−159.8 (2)
C1C—C6C—O1C—Zn7	5.3 (4)	O3C—Zn7—N1C—C8C	−44.8 (5)
C5C—C6C—O1C—Zn7	−174.42 (17)	O7W—Zn7—N1C—C8C	93.1 (2)
C1C—C6C—O1C—Zn3	−177.1 (2)	N2C—Zn7—N1C—C8C	−22.5 (2)
C5C—C6C—O1C—Zn3	3.2 (3)	C11B—C10B—N2B—C9B	−179.4 (2)
O3C—Zn7—O1C—C6C	176.1 (2)	C11B—C10B—N2B—Zn6	4.1 (4)
O7W—Zn7—O1C—C6C	78.6 (2)	C8B—C9B—N2B—C10B	153.7 (2)
N2C—Zn7—O1C—C6C	−85.9 (2)	C8B—C9B—N2B—Zn6	−29.4 (3)
N1C—Zn7—O1C—C6C	−14.2 (2)	O1B—Zn6—N2B—C10B	103.6 (2)
O3C—Zn7—O1C—Zn3	−1.32 (11)	O3B—Zn6—N2B—C10B	2.4 (2)
O7W—Zn7—O1C—Zn3	−98.86 (10)	O6W—Zn6—N2B—C10B	−93.8 (2)
N2C—Zn7—O1C—Zn3	96.71 (13)	N1B—Zn6—N2B—C10B	179.5 (2)
N1C—Zn7—O1C—Zn3	168.42 (11)	O1B—Zn6—N2B—C9B	−73.1 (2)
O4A—Zn3—O1C—C6C	116.39 (16)	O3B—Zn6—N2B—C9B	−174.25 (18)
O2C—Zn3—O1C—C6C	−3.18 (16)	O6W—Zn6—N2B—C9B	89.56 (19)
O4B—Zn3—O1C—C6C	−119.88 (16)	N1B—Zn6—N2B—C9B	2.88 (18)
O3B—Zn3—O1C—C6C	−50.8 (3)	C11A—C10A—N2A—C9A	−177.2 (3)
O4A—Zn3—O1C—Zn7	−65.78 (11)	C11A—C10A—N2A—Zn5	−5.2 (5)
O2C—Zn3—O1C—Zn7	174.65 (11)	C8A—C9A—N2A—C10A	−165.0 (3)
O4B—Zn3—O1C—Zn7	57.95 (10)	C8A—C9A—N2A—Zn5	22.0 (3)
O3B—Zn3—O1C—Zn7	126.99 (19)	O1A—Zn5—N2A—C10A	−99.6 (3)
C4A—C5A—O2A—Zn1	160.9 (3)	O3A—Zn5—N2A—C10A	2.1 (3)
C6A—C5A—O2A—Zn1	−17.8 (3)	O5W—Zn5—N2A—C10A	97.0 (3)
O4C—Zn1—O2A—C5A	−81.6 (2)	N1A—Zn5—N2A—C10A	−169.3 (3)
O4D—Zn1—O2A—C5A	105.5 (2)	O1A—Zn5—N2A—C9A	72.7 (3)
O1A—Zn1—O2A—C5A	17.65 (19)	O3A—Zn5—N2A—C9A	174.4 (2)
O3D—Zn1—O2A—C5A	−178.6 (2)	O5W—Zn5—N2A—C9A	−90.7 (2)
C5D—C6D—O2D—Zn8	−1.8 (4)	N1A—Zn5—N2A—C9A	2.9 (2)
C1D—C6D—O2D—Zn8	−179.73 (17)	C11C—C10C—N2C—C9C	−177.4 (3)
C5D—C6D—O2D—Zn4	168.5 (2)	C11C—C10C—N2C—Zn7	0.8 (4)
C1D—C6D—O2D—Zn4	−9.4 (3)	C8C—C9C—N2C—C10C	−150.6 (3)
O3D—Zn8—O2D—C6D	172.39 (19)	C8C—C9C—N2C—Zn7	31.0 (3)
O8W—Zn8—O2D—C6D	−90.4 (2)	O1C—Zn7—N2C—C10C	−109.0 (2)
N3D—Zn8—O2D—C6D	2.6 (2)	O3C—Zn7—N2C—C10C	−8.1 (2)
N4D—Zn8—O2D—C6D	72.3 (2)	O7W—Zn7—N2C—C10C	87.5 (2)
O3D—Zn8—O2D—Zn4	3.13 (11)	N1C—Zn7—N2C—C10C	176.1 (3)
O8W—Zn8—O2D—Zn4	100.31 (10)	O1C—Zn7—N2C—C9C	69.3 (2)
N3D—Zn8—O2D—Zn4	−166.66 (11)	O3C—Zn7—N2C—C9C	170.2 (2)
N4D—Zn8—O2D—Zn4	−96.94 (15)	O7W—Zn7—N2C—C9C	−94.2 (2)
O1D—Zn4—O2D—C6D	11.23 (16)	N1C—Zn7—N2C—C9C	−5.6 (2)
O4B—Zn4—O2D—C6D	−103.68 (16)	O2—C4—N2—C6	0.0 (5)
O4C—Zn4—O2D—C6D	130.05 (16)	O2—C4—N2—C5	−180.0 (3)
O3C—Zn4—O2D—C6D	62.6 (3)	C5D—C7D—N3D—C8D	−173.7 (3)
O1D—Zn4—O2D—Zn8	−177.71 (11)	C5D—C7D—N3D—Zn8	−3.4 (4)
O4B—Zn4—O2D—Zn8	67.37 (11)	C9D—C8D—N3D—C7D	124.0 (3)
O4C—Zn4—O2D—Zn8	−58.89 (11)	C9D—C8D—N3D—Zn8	−47.9 (3)
O3C—Zn4—O2D—Zn8	−126.3 (3)	O2D—Zn8—N3D—C7D	−0.1 (3)
C4B—C5B—O2B—Zn2	178.2 (2)	O3D—Zn8—N3D—C7D	−122.2 (4)
C6B—C5B—O2B—Zn2	0.0 (3)	O8W—Zn8—N3D—C7D	107.3 (3)
O4D—Zn2—O2B—C5B	100.08 (19)	N4D—Zn8—N3D—C7D	−144.5 (3)
O4A—Zn2—O2B—C5B	−91.9 (2)	O2D—Zn8—N3D—C8D	171.07 (18)
O1B—Zn2—O2B—C5B	−1.00 (19)	O3D—Zn8—N3D—C8D	49.0 (5)
O3A—Zn2—O2B—C5B	−166.98 (19)	O8W—Zn8—N3D—C8D	−81.54 (18)
C4C—C5C—O2C—Zn3	179.2 (2)	N4D—Zn8—N3D—C8D	26.61 (17)
C6C—C5C—O2C—Zn3	−1.8 (3)	O3—C7—N3—C9	−179.8 (6)
O4A—Zn3—O2C—C5C	−99.03 (18)	O3—C7—N3—C8	0.4 (10)
O4B—Zn3—O2C—C5C	92.67 (18)	O4—C10—N4—C12	−5.1 (6)
O1C—Zn3—O2C—C5C	2.66 (17)	O4—C10—N4—C11	−177.7 (3)
O3B—Zn3—O2C—C5C	169.36 (17)	C11D—C10D—N4D—C9D	−178.4 (2)
C11D—C16D—O3D—Zn8	7.4 (3)	C11D—C10D—N4D—Zn8	2.3 (4)
C15D—C16D—O3D—Zn8	−172.64 (16)	C8D—C9D—N4D—C10D	154.8 (2)
C11D—C16D—O3D—Zn1	158.5 (2)	C8D—C9D—N4D—Zn8	−25.7 (3)
C15D—C16D—O3D—Zn1	−21.5 (2)	O2D—Zn8—N4D—C10D	106.8 (2)
O2D—Zn8—O3D—C16D	−151.98 (18)	O3D—Zn8—N4D—C10D	4.2 (2)
O8W—Zn8—O3D—C16D	99.72 (18)	O8W—Zn8—N4D—C10D	−90.5 (2)
N3D—Zn8—O3D—C16D	−30.7 (5)	N3D—Zn8—N4D—C10D	179.7 (2)
N4D—Zn8—O3D—C16D	−8.77 (18)	O2D—Zn8—N4D—C9D	−72.6 (2)
O2D—Zn8—O3D—Zn1	60.66 (10)	O3D—Zn8—N4D—C9D	−175.21 (18)
O8W—Zn8—O3D—Zn1	−47.64 (10)	O8W—Zn8—N4D—C9D	90.09 (18)
N3D—Zn8—O3D—Zn1	−178.1 (3)	N3D—Zn8—N4D—C9D	0.26 (18)
N4D—Zn8—O3D—Zn1	−156.13 (10)	O5—C13—N5—C15	175.7 (3)
O4C—Zn1—O3D—C16D	153.81 (15)	O5—C13—N5—C14	3.4 (5)
O2A—Zn1—O3D—C16D	−97.43 (15)	O6—C19—N6—C18	6.6 (6)
O4D—Zn1—O3D—C16D	27.30 (14)	O6—C19—N6—C17	178.7 (3)
O1A—Zn1—O3D—C16D	−40.5 (3)		

### Hydrogen-bond geometry (Å, °)

**Table:**

*D*—H···*A*	*D*—H	H···*A*	*D*···*A*	*D*—H···*A*
O8W—H81W···O4	0.85	1.89	2.720 (3)	163
O8W—H82W···O2A	0.85	1.70	2.545 (3)	172
O7W—H21W···O5	0.85	1.84	2.677 (3)	167
O7W—H22W···O1D	0.85	1.76	2.604 (3)	173
O6W—H61W···O2	0.85	1.88	2.723 (3)	174
O6W—H62W···O2C	0.85	1.73	2.569 (3)	170
O5W—H51W···O6	0.85	1.89	2.731 (3)	171
O5W—H52W···O2B	0.85	1.77	2.612 (2)	171
C13A—H13A···O4^i^	0.93	2.52	3.339 (4)	148
C10D—H10D···O1	0.93	2.47	3.294 (4)	147
C13—H13···O4B	0.93	2.59	3.514 (4)	175

Symmetry codes: (i) *x*+1, *y*, *z*.
